# Probing Site‐Selective Conjugation Chemistries for the Construction of Homogeneous Synthetic Glycodendriproteins

**DOI:** 10.1002/cbic.202200020

**Published:** 2022-04-01

**Authors:** Isidro Cobo, M. Isabel Matheu, Sergio Castillón, Benjamin G. Davis, Omar Boutureira

**Affiliations:** ^1^ Departament de Química Analítica i Química Orgànica Facultat de Química Universitat Rovira i Virgili C/ Marcel⋅lí Domingo 1 43007 Tarragona Spain; ^2^ Department of Chemistry Chemistry Research Laboratory University of Oxford Mansfield Road Oxford OX1 3TA UK; ^3^ Department of Pharmacology University of Oxford Mansfield Road Oxford OX1 3QT UK; ^4^ The Rosalind Franklin Institute, Harwell Campus Didcot, Oxfordshire OX11 0FA UK

**Keywords:** bioconjugations, bioorthogonal reactions, dendrimers, glycoproteins, protein modifications

## Abstract

Methods that site‐selectively attach multivalent carbohydrate moieties to proteins can be used to generate homogeneous glycodendriproteins as synthetic functional mimics of glycoproteins. Here, we study aspects of the scope and limitations of some common bioconjugation techniques that can give access to well‐defined glycodendriproteins. A diverse reactive platform was designed *via* use of thiol‐Michael‐type additions, thiol‐ene reactions, and Cu(I)‐mediated azide‐alkyne cycloadditions from recombinant proteins containing the non‐canonical amino acids dehydroalanine, homoallylglycine, homopropargylglycine, and azidohomoalanine.

## Graphical abstract

**Homogeneous glycodendriproteins**: A comparative reactive platform was designed *via* use of C−S bond‐forming reactions (unprecedented for this family of glycoconjugates) and *via* triazoles (in two orientations), from recombinant proteins containing non‐canonical amino acids (dehydroalanine, homoallylglycine, homopropargylglycine, and azidohomoalanine) that can give access to well‐defined glycodendriproteins as synthetic functional mimics of *N*‐linked glycoproteins.

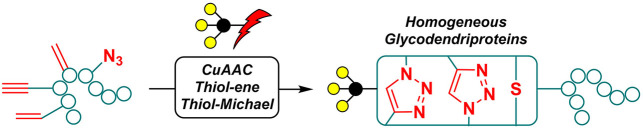

## Introduction

**Figure 1:**
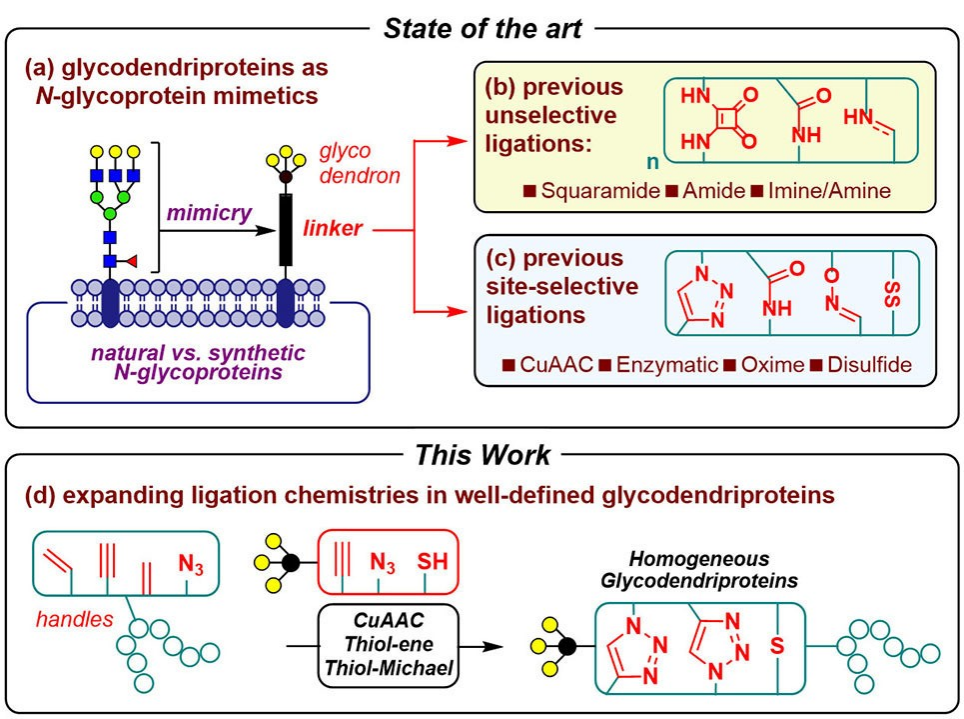
(a) Synthetic glycodendriproteins as putative *N*‐glycoprotein mimetics. Glycodendriproteins previously prepared from (b) unselective and (c) site‐selective conjugation methods. (d) *This work*: expanding ligation protocols in chemically‐defined glycodendriproteins.

The use of synthetic glycosylated macromolecules or glycoconjugates, such as glycodendrimers and glyconanoparticles, can functionally mimic aspects of the sugar display of glycoproteins and glycolipids that decorate the outer surface of mammalian cells. For example, the blocking by decoys of carbohydrate‐protein(lectin) interactions can represent an attractive anti‐infective strategy for targeting pathogens.^[1]^ Among glycoconjugates, synthetic glycoproteins^[2]^ and particularly glycodendriproteins, resulting from the attachment of multivalent, antennary‐like carbohydrate epitopes to a precise site of a protein scaffold (Figure 1a), have emerged as a class of mimics of naturally occurring *N*‐linked glycoproteins with additional implications in vaccine design,^[3]^ the development of bacterial/viral aggregation inhibitors,[^4^, ^5^, ^6^] ligands for the mannose‐6‐phosphate (M6PR)^[7]^ and asialoglycoprotein (ASGPR)^[8]^ receptors, and as glycomimetics of insulin,^[9]^ human growth hormone, and the Fc region of human IgG.^[10]^

Whilst multivalent glycoprotein inhibitors of pathogen adhesion can be prepared by the non‐selective attachment of dendrimeric glycans to proteins *via* standard amide, squaramide, or imine/amine formation,^[11]^ recent advances in selective chemical protein modification^[12]^ have allowed the precise attachment of multivalent carbohydrate moieties to predetermined sites of proteins using biologically‐compatible reactions to generate single, well‐defined glycodendriprotein glycoforms (Figure 1b,c). However, despite this progress, such methods remain scarce and have employed diverse linkages, including those derived enzymatically (amides),^[9]^ those that are potentially cleavable/reversible (disulfides[^4^, ^5^] and oximes^[10]^), and those that are stable under physiological conditions (*e. g*., 1,4‐triazole linkages derived from Cu(I)‐mediated azide‐alkyne cycloadditions (CuAAC) with the alkyne tag homopropargylglycine (Hpg)^[6]^ or *N6*‐[(2‐propynyloxy)carbonyl]‐L‐lysine (Lys(PA))^[7]^).

The aim of this proof‐of‐principle study is to comparatively evaluate the efficiency of some common, site‐selective protein chemistries that could yield well‐defined glycodendriproteins and so therefore may prove attractive in the design of putative synthetic protein therapeutics (synthetic biologics). By using comparable, representative tri‐antennary, tri‐galactosyl (β‐D‐Gal)_3_ carbohydrate dendron motifs, each equipped with corresponding reactive handles, we explored the generation of a series of recombinant glycodendriproteins using an approach of ‘tag‐and‐modify’ with several tag types.^[13]^ These allowed the testing of thiol‐Michael‐type additions to dehydroalanine (Dha)‐tagged proteins,^[14]^ thiol‐ene^[15]^ radical additions at homoallylglycine (Hag) sites to generate thio‐ether linkages,^[27]^ and Cu(I)‐mediated azide‐alkyne cycloadditions to access 1,4‐triazole linkages in two orientations, *via* azidohomoalanine (Aha) and homopropargylglycine (Hpg) tags (Figure 1d).[^6^, ^33^]

## Results and Discussion

### Synthesis of glycodendron reagents

**Scheme 1:**
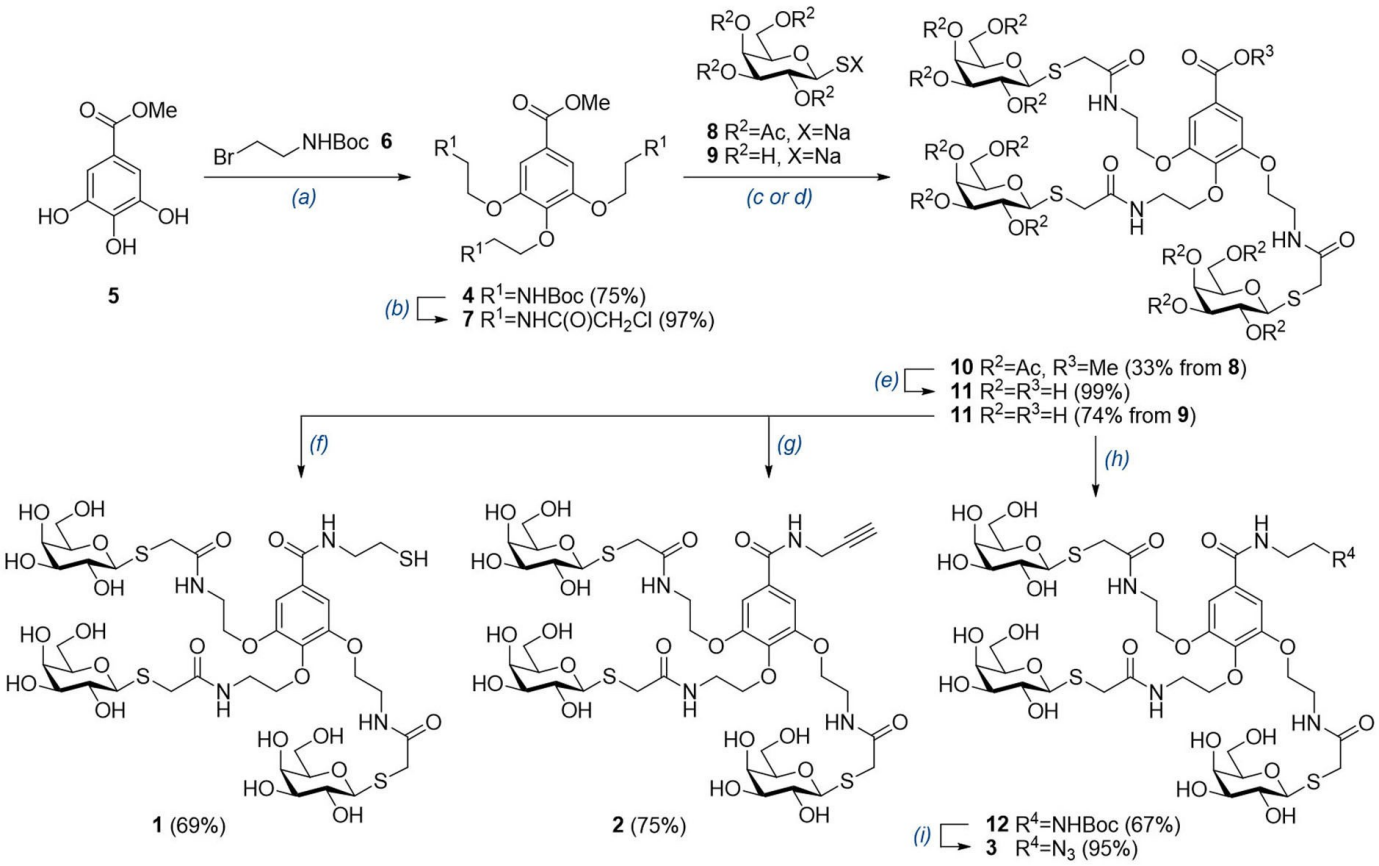
Synthesis of tri‐antennary glycodendron reagents **1**–**3**. *Reagents and conditions*: (a) dry K_2_CO_3_, 10 mol% TBAI, dry DMF, 80 °C, 6 d; (b) (*i*) dry CH_2_Cl_2_, 4 M HCl in dioxane, rt, 2 h, (*ii*) chloroacetyl chloride, NaHCO_3_, 2 : 1 Et_2_O/H_2_O, 0 °C to rt, 7 d; (c) **8**, dry DMF, rt, 3 d; (d) (*i*) **9**, dry DMF, rt, 24 h, (*ii*) 1 : 10 1 N NaOH (aq.)/EtOH, rt, 22 h; (e) (*i*) 1 N NaOMe, dry MeOH, rt, 1 h, (*ii*) 1 : 10 1 N NaOH (aq.)/EtOH, rt, 24 h; (f) (*i*) cystamine, HATU, DIPEA, dry DMF, 50 °C, 3 d, (*ii*) PBu_3_, H_2_O, rt, 2 h. (g) propargylamine hydrochloride, HATU, DIPEA, dry DMF, 45 °C, 27 h; (h) *N*‐Boc‐ethylenediamine, HATU, DIPEA, dry DMF, 45 °C, 27 h; (i) (*i*) 1 : 2 Me_2_S/TFA, 0 °C, 3 h, (*ii*) 0.4 M TfN_3_ in CH_2_Cl_2_, 10 mol% CuSO_4_, DMAP, MeOH, 0 °C to rt, 19 h. Boc=*tert*‐butoxycarbonyl, TBAI=tetrabutylammonium iodide, DMF=*N*,*N*‐dimethylformamide, HATU=1‐[bis(dimethylamino)methylene]‐1*H*‐1,2,3‐triazolo[4,5‐b]pyridinium 3‐oxide hexafluorophosphate, DIPEA=*N,N*‐diisopropylethylamine, TFA=trifluoroacetic acid, DMAP=4‐dimethylaminopyridine.

Model tri‐β‐D‐galactosyl‐containing (β‐D‐Gal)_3_ glycodendrons **1**–**3** derived from a 3,4,5‐*tris*(2‐aminoethoxy)benzoic acid core and equipped with appropriate reactive handles (thiol, propargyl, and azide) were designed and synthesized as simple mimics of the asymmetric carbohydrate display observed in tri‐antennary *N*‐linked glycoproteins (Scheme 1).^[16]^ These structures possess significant rigidity and useful distances between their β‐D‐Gal tip sugars, features that can prove favourable for multivalent ligand display.[^17^, ^18^] Indeed, it has been noted that glycodendrimers can mimic the non‐reducing termini as well as some secondary interactions using only imperfect structural analogues^[19]^ of the branched carbohydrates found in glycoproteins, without the necessity of presenting the whole, synthetically‐challenging, natural complex oligosaccharide.^[20]^ In addition, we selected thioglycosides as the glycan motifs in these glycodendrons as these typically confer greater stability under both basic and acidic aqueous conditions, as well as resistance to enzymatic hydrolysis.^[21]^ Moreover, such thioglycoside mimetics, together with other chalcogen derivatives such as selenoglycosides, can maintain the intrinsic binding properties of the glycan towards the corresponding protein receptor (lectin).^[22]^

We first prepared extended scaffold **4** as a key intermediate. After some preliminary attempts and reaction optimization (Supporting Information (SI), Schemes S1 and S2), **4** was obtained in 75 % yield from **5** and **6** as previously described by Brouwer *et al*.^[17]^ Boc deprotection using 4 M HCl in dioxane and subsequent treatment of the resulting trihydrochloride salt with chloroacetyl chloride and NaHCO_3_ using a biphasic 2 : 1 Et_2_O/H_2_O solvent system afforded derivative **7** (97 %). Boc removal using standard trifluoroacetic acid (TFA) and the use of chloroacetic anhydride as *N*‐acylating reagent led to lower overall yields (Supporting Information, Scheme S3). Next, the incorporation of the non‐reducing β‐D‐galactose moiety (β‐D‐Gal) was first attempted using *O*‐acetyl protected thioglycoside sodium salt **8** ^[34]^ in dry DMF. However, **10** was obtained in only 33 % yield, and despite quantitative subsequent Zemplén deacetylation and methyl ester hydrolysis to **11** (99 %), the reduced overall yield hampered the utilization of this route using protecting groups. Thus, an alternative protecting‐group‐free route was explored (Supporting Information, Table S1). Treatment of gallic acid core **7** with β‐thiogalactoside sodium salt **9** ^[35]^ followed by methyl ester hydrolysis using aqueous NaOH in EtOH allowed the ready preparation of common glycodendron reagent precursor **11** in a superior 74 % yield over two steps. Next, using this divergent intermediate, appropriate reactive handles (thiol, propargyl, and azide) were introduced through amide‐coupling protocols (Scheme 1). A reactive thiol was incorporated by treating **11** and cystamine with HATU and DIPEA in dry DMF at 50 °C to afford a disulfide intermediate, which was subsequently reduced *in situ* to **1** (69 %) with PBu_3_ in water at room temperature for 2 h. Similarly, an alkynyl group was incorporated to obtain reagent **2** (75 %) after stirring a mixture of **11**, propargylamine hydrochloride, HATU, and DIPEA in dry DMF at 45 °C for 27 h. Finally, reactive azide was incorporated following a 3‐step procedure. Similarly to propargyl **2**, amide coupling with *N*‐Boc‐ethylenediamine afforded **12** in 67 % yield. Subsequent Boc deprotection with 1 : 2 Me_2_S/TFA followed by diazo transfer to the resulting primary amine led to azide **3** in 95 % yield over two steps.

### Glycodendriprotein construction

**Scheme 2:**
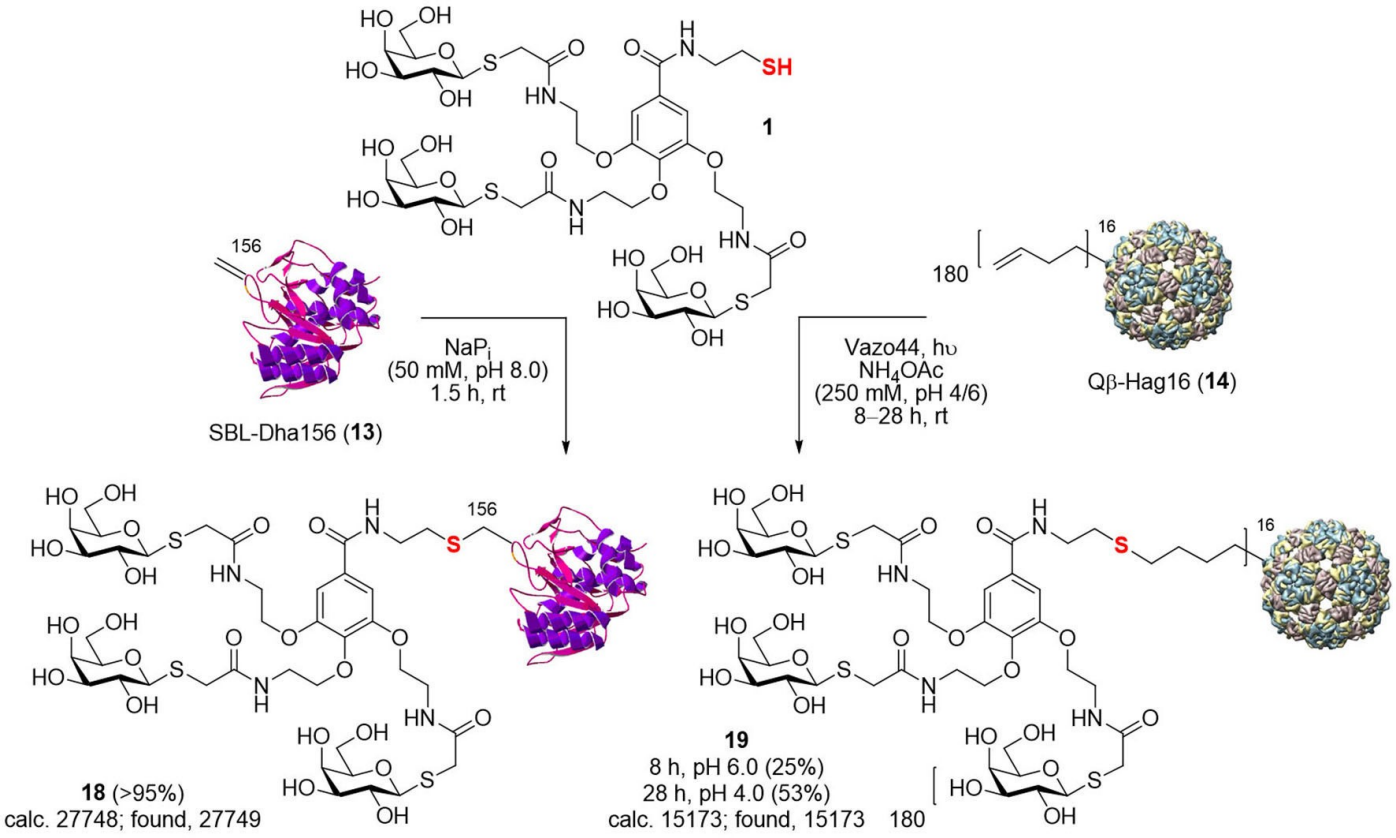
Synthesis of *S*‐linked glycodendriproteins **18** and **19** from SBL‐Dha156 (**13**) and Qβ‐Hag16 (**14**), respectively. Note that whilst reactions were conducted under non‐denatured conditions as shown masses, the intact protein masses shown for Qβ refer to monomer following denaturation to individual monomers for ESI‐MS analysis.

The next step of the proposed strategy involved the use of these synthesized tri‐antennary glycodendron reagents **1**–**3** to chemical modify a series of protein substrates bearing appropriate reactive tags; Dha **13**[^23^, ^24^, ^25^, ^26^] (here chemically generated from Cys), Hag **14**,[^23^, ^27^] Aha **15**,^[28]^ **16**,[^25^, ^28^] and Hpg **17** ^[29]^ (the latter three were all generated through sense‐codon reassignment of Met exploiting Met‐auxotroph‐mediated expression). In order to rapidly scope the generality of our tested methods to access well‐defined glycodendriproteins, we used a multivariate selection of prototypical protein scaffolds featuring different residue sites/microenvironments, protein folds, as well as possessing different functional measures (catalytic activity or structural/self‐assembling properties) of outcome. We first explored thiol‐conjugate‐addition chemistry, an approach absent from previous protocols for glycodendriprotein generation, using a single Dha protein mutant of the serine protease subtilisin from *Bacillus lentus* (SBL), a representative three‐layer α/β‐Rossman‐fold protein with catalytic activity, quantitatively obtained from the corresponding Cys precursor using standard bisalkylation‐elimination protocols.^[30]^ The identity, purity, and stability of the resulting glycodendriproteins was established by liquid chromatography electrospray ionization mass spectrometry (LC‐ESI‐MS) and SDS polyacrylamide gel electrophoresis (SDS‐PAGE) (Supporting Information, Figures S1‐*‐*S16). Thus, after generation from SBL‐Cys156, incubation of SBL‐Dha156 (**13**) with **1** in 50 mM sodium phosphate buffer (NaP_i_) at pH 8.0 afforded pure, synthetic glycodendriprotein **18** in >95 % conversion after 1.5 h at room temperature as determined by LC‐ESI‐MS (Scheme 2, left panel). Although CuAAC reactions are somewhat more established in the limited examples of glycodendriprotein generation, the use of this alternative method provides potential expansion of scope and also allows potential access to alternative reaction scoping from the same glycodendron (here thiol **1**) type.

**Scheme 3:**
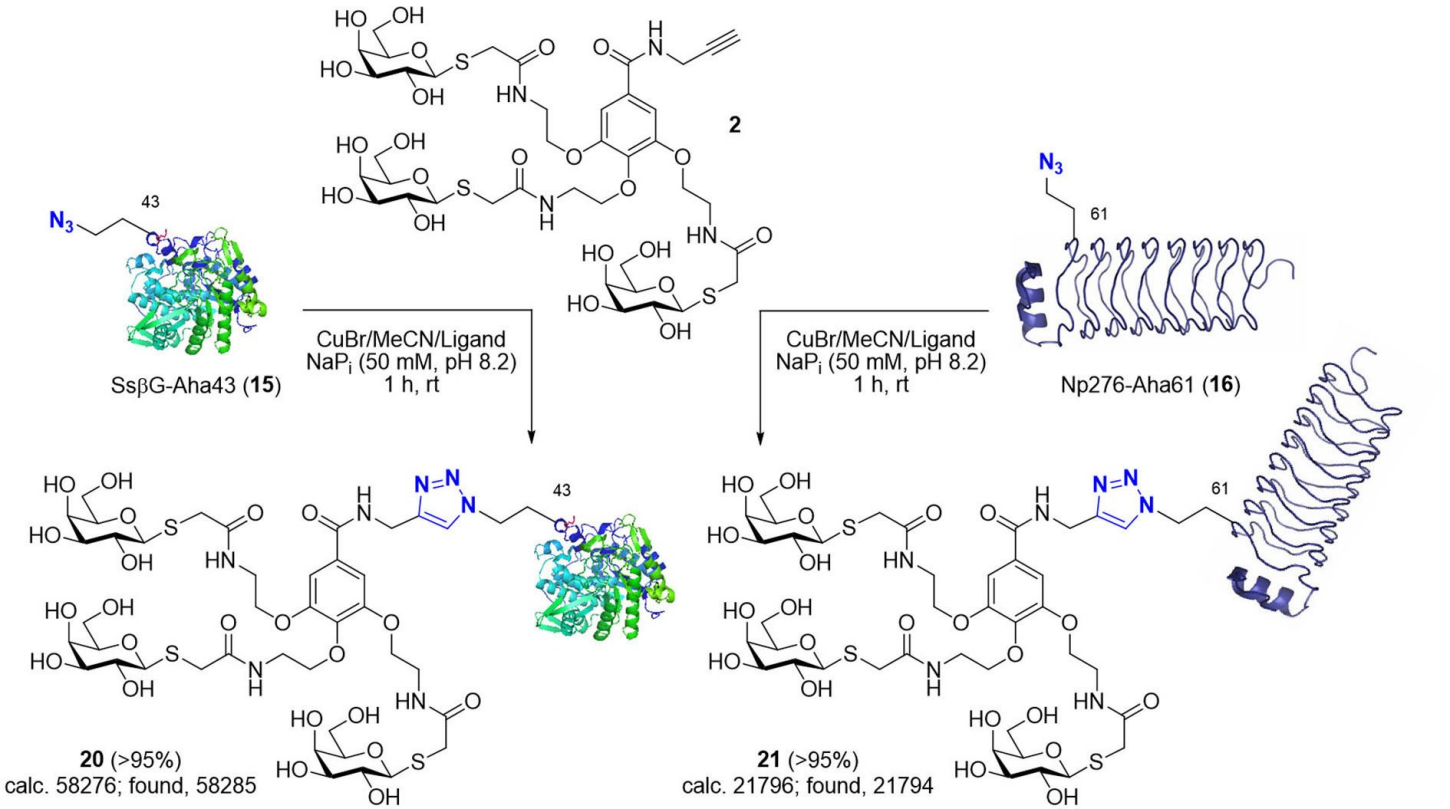
Synthesis of *triazole*‐linked glycodendriproteins **20** and **21** from *S*sβG‐Aha43 (**15**) and Np276‐Aha61 (**16**), respectively.

**Scheme 4:**
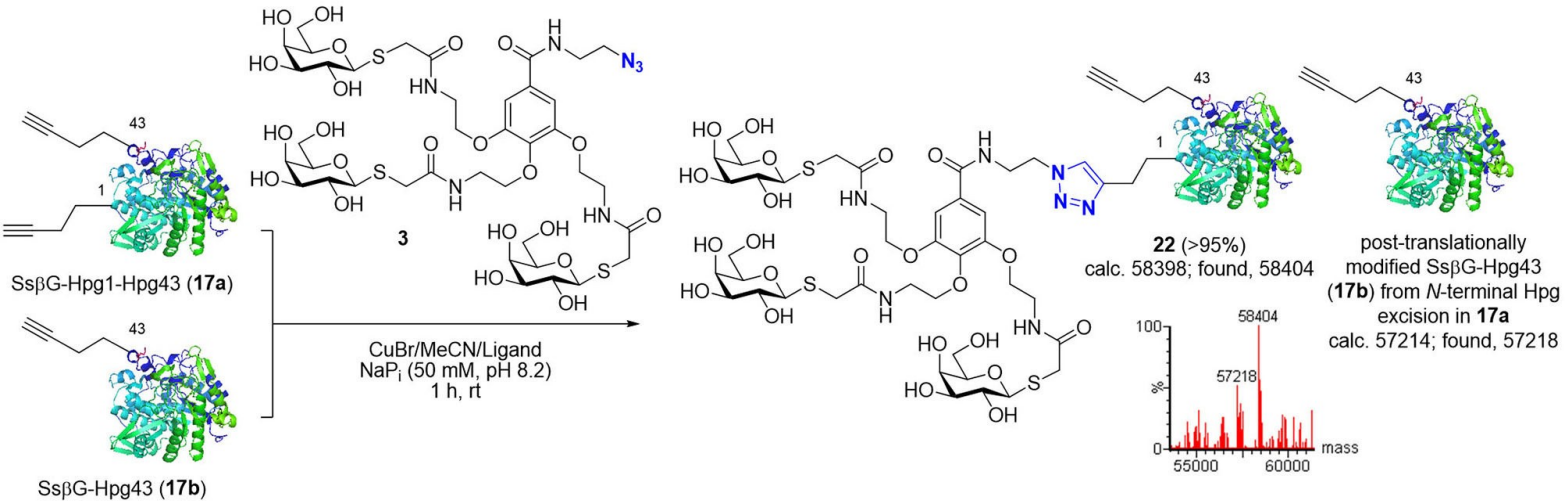
Comparative reactivity in the attempted generation of *triazole*‐linked glycodendriprotein **22** from a mixture of *S*sβG‐Hpg1‐Hpg43/‐Hpg43 (**17 a**/**b**), as indicated by crude intact protein MS of reaction mixture. On the basis of the apparent reaction only of **17 a**, Hpg at site 43 appears unreactive (albeit on basis of analysis with low signal to noise), in contrast to Aha at site 43 (see Scheme 3).

Indeed, next, thiol*‐*ene radical addition/ligation was explored as alternative/complementary thiol chemistry to this previous thiol‐Michael addition. Here, we explored the use of homomultimer, virus‐like bacteriophage particle Qβ **14**, which self‐assembles from 180 monomers, equipped with a Hag tag (Scheme 2, right panel).[^23^, ^27^] This icosahedral protein platform provides greatly differing dimensions (core diameter ∼28 nm), and so potentially reactivities. It has also allowed the prior construction of multivalent systems with enhanced function (*e. g*., viral mimicry^[6]^). Whilst direct comparison in this different protein scaffold would be inappropriate, use of the same thiol glycodendron **1** in thiol*‐*ene reaction Qβ‐Hag16 (**14**) was sluggish, and final glycodendriprotein nanoparticle **19** was obtained in only 25 % conversion at pH 6.0 and 53 % conversion at pH 4.0 (500 equiv. of glycodendron reagent **1**, after 8 and 28 h at room temperature, respectively);^[23]^ increased equivalents of **1** resulted in only similar conversion levels. Reactions carried out at pH 6.0 provided more homogeneous product (as judged, for example, by MS spectrum signal‐to‐noise (S/N)) than those at pH 4.0, albeit with lower conversions. Moreover, prolonged reaction times did not substantially improve the conversion. Next, to broaden the scope of reactions tested and protein scaffolds/sites/residue microenvironments, we explored well‐established Cu(I)‐mediated azide‐alkyne cycloadditions (CuAAC) using a variety of protein scaffolds with alkyne and azide tags; *S*sβG‐Aha43 (**15**), *S*sβG‐Hpg1‐Hpg43/‐Hpg43 (**17 a**/**b**) (as an example of a generic β‐galactosidase, in an αβ‐fold TIM barrel, that has been previously used to create synthetic glycoprotein probes for both *in vitro* and *in vivo* applications)^[33]^ and Np276‐Aha61 (**16**) (in an all‐β‐helix, β‐fold pentapeptide repeat protein scaffold from *Nostoc punctiforme*, fusion protein 275/276 also known as Npβ) (Schemes 3 and 4). In this way, this allowed not only site‐exploration within varied scaffolds with differing secondary structural features (β dominant *vs*. αβ mixed) but again also protein function variation (*e. g*., catalytic activity). Triazole‐linked products **20**, **21** were generated efficiently in conversions of >95 % upon incubation with propargyl **2** glycodendron reagent in 50 mM NaP_i_ at pH 8.2 for 1 h at room temperature as determined by LC‐ESI‐MS (Scheme 3). By contrast, under the same conditions, reaction of azide **3** with *S*sβG‐Hpg1‐Hpg43 (**17 a**) was more sluggish. Notably, when azide **3** was used to modify a mixture of *S*sβG‐Hpg1‐Hpg43/‐Hpg43 (**17 a**/**b**), crude product **22** was generated consistent with regioselective monomodification only at a more accessible position 1 (*i. e*., reaction only of *S*sβG‐Hpg1‐Hpg43 and not of *S*sβG‐Hpg43). Such regioselectivity in the use of CuAAC on proteins is consistent with previous observations (Scheme 4).^[29]^ This apparent dependency of reaction conversion upon protein site location (*i. e*., regioselectivity) mirrors previous observations[^29^, ^31^, ^33^] where correlation is observed with a combination of the intrinsic reactivity of the tag‐reactant pair as well as the protein residue accessibility. The possible additional roles of residue microenvironment (*e. g*., charges, polar/hydrophobic interactions, etc.) may well also play a role but have typically proven less important in our hands. As a consequence, the presence at the same site of different reactive tags (Hpg43 *vs*. Aha43) or the same reactive tag at different site (Hpg1 *vs*. Hpg43) may react differently. This was previously rationalized according to a heuristic model (termed ‘reactive accessibility’, RA) to evaluate and predict site reactivity.^[33]^ This model correlates the reactivity observed with predicted measures of protein residue accessibility^[32]^ and is able to, in turn, guide the control of reaction conditions to achieve regioselective modification at a variety of protein sites.[^31^, ^33^] The apparent contrast in the reactivity between Aha and Hpg at the same site 43 in protein *S*sβG reinforces the importance of evaluating CuAAC reaction ‘orientations’ when deciding the choice of reactive handle location in two partners to be conjugated and the preferential use in our hands of Aha as a tag for less accessible sites and in proteins. Our observations (here and previously) consistently suggest lower protein reactivity in Hpg‐tagged proteins compared to their Aha‐tagged counterparts.[^6^, ^29^, ^33^]

## Conclusion

In summary, a brief survey of the site‐selective attachment of simple, multivalent (β‐D‐Gal)_3_ dendrons to generate glycodendriproteins as *N*‐linked glycoprotein mimics suggests that although well‐defined, highly‐valent structures can be generated through other methods,^[6]^ those based on the use of Dha tags (for C−S‐bond formation) and Aha tags (for triazole formation) may be the most applicable to the ready formation of well‐defined constructs. A qualitative comparison of the use of glycodendrons to modify proteins both here and previously^[6]^ suggests an apparent order of utility in these systems as follows, *tags‐via‐linkage*: Dha‐*via*‐C−S [Dha+R‐SH]∼Aha‐*via*‐triazole [Aha+R−C≡CH]>Hpg‐*via*‐triazole [Hpg+R‐N_3_]>Hag‐*via*‐C−S [Hag+R−S⋅]. With regard to application, it has been previously shown that the conjugation of glycodendron reagents to proteases, such as SBL used here, enables targeted protein degradation; this has been applied to, for example, bind and degrade bacterial adhesins.^[5]^ As such, not only might glycodendriprotein glycoconjugates allow development of anti‐infective therapeutics (by both direct blocking^[6]^ and degradation^[5]^) but also other potentially broader clinical applications that may exploit the selective degradation of other sugar‐binding proteins. Work to this goal is currently under investigation in our laboratories.

## Experimental Section

**General remarks**: Proton (^1^H NMR) and carbon (^13^C NMR) nuclear magnetic resonance spectra were recorded on a Varian Mercury spectrometer (400 MHz for ^1^H) and (100.6 MHz for ^13^C) or a Bruker AVII500 spectrometer (500 MHz for ^1^H) and (125.8 MHz for ^13^C). NMR spectra were assigned using COSY, DEPT 135, HSQC, HMBC, and NOESY and are subjective. All chemical shifts are quoted on the *δ* scale in ppm using the residual solvent as the internal standard (^1^H NMR: CDCl_3_=7.26, DMSO‐*d*_6_=2.50, D_2_O=4.79 and ^13^C NMR: CDCl_3_=77.0; DMSO‐*d*_6_=39.5). Coupling constants (*J*) are reported in Hz with the following splitting abbreviations: s=singlet, d=doublet, t=triplet, q=quartet, quin=quintet and app=apparent. Melting points (mp) were recorded on a Leica Galen III hot stage microscope equipped with a Testo 720 thermocouple probe and are uncorrected. Infrared (IR) spectra were recorded on a Bruker Tensor 27 Fourier Transform (FT) spectrophotometer using thin films on NaCl plates for liquids and oils and KBr discs for solids and crystals. Absorption maxima (*ν*_max_) are reported in wavenumbers (cm^−1^). Elemental analyses (C, H, N, and S) were performed with a Carlo Erba EA 1108 Analyser. Optical rotations were measured on a Perkin‐Elmer 241 polarimeter with a path length of 1.0 dm and are reported with implied units of 10^−1^ deg cm^2^ g^−1^. Concentrations (*c*) are given in g/100 mL. Low resolution mass spectra (LRMS) were recorded on a Waters Micromass LCT Premier TOF spectrometer using electrospray ionization (ESI) and high resolution mass spectra (HRMS) were recorded on a Bruker MicroTOF ESI mass spectrometer. Nominal and exact *m/z* values are reported in Daltons (Da). Thin layer chromatography (TLC) was carried out using Merck aluminium backed sheets coated with 60F_254_ silica gel. Visualization of the silica plates was achieved using a UV lamp (λ_max_=254 nm) and/or ammonium molybdate (5 % in 2 M H_2_SO_4_) and/or potassium permanganate (5 % KMnO_4_ in 1 M NaOH with 5 % K_2_CO_3_). Flash column chromatography was carried out using BDH 40–63 μm silica gel (VWR). Mobile phases are reported in relative composition (*e. g*., 1 : 2 : 4 H_2_O/*i*PrOH/EtOAc v/v/v). Anhydrous solvents were purchased from Fluka or Acros. All other solvents were used as supplied (Analytical or HPLC grade), without prior purification. Distilled water was used for chemical reactions and Milli‐QR purified water for protein manipulations. All reactions using anhydrous conditions were performed using flame‐dried apparatus under an atmosphere of argon or nitrogen. ‘Petrol’ refers to the fraction of light petroleum ether boiling in the range 40–60 °C. Brine refers to a saturated solution of sodium chloride. Anhydrous magnesium sulfate (MgSO_4_) was used as drying agent after reaction work‐up, as indicated.

**Protein liquid chromatography‐mass spectrometry analysis**: Liquid chromatography‐mass spectrometry (LC–MS) was performed on a Micromass LCT (ESITOF‐MS) coupled to a Waters Alliance 2790 HPLC using a Phenomenex Jupiter C4 column (250×4.6 mm×5 μm). Water (solvent A) and acetonitrile (solvent B), each containing 0.1 % formic acid, were used as the mobile phase at a flow rate of 1.0 mL min^−1^. The gradient was programmed as follows: 95 % A (5 min isocratic) to 100 % B after 15 min then isocratic for 5 min. The electrospray source was operated with a capillary voltage of 3.2 kV and a cone voltage of 25 V (35 V for β‐galactosidase (*S*sβG)). Nitrogen was used as the nebulizer and desolvation gas at a total flow of 600 L h^−1^. Spectra were calibrated using a calibration curve constructed from a minimum of 17 matched peaks from the multiply charged ion series of equine myoglobin obtained at a cone voltage of 25 V. A typical analysis of a conjugation reaction by LC‐ESI‐MS is described as follows. Briefly, integration of the region containing all protein (both starting material and products) in the total ion chromatogram afforded the combined ion series. Deconvoluted total mass spectrum was reconstructed from the ion series using the MaxEnt algorithm preinstalled on MassLynx software (v. 4.0 from Waters) according to the manufacturer's instructions. Identical analyses were carried out for all the conjugation reactions performed in this work.

**Protein SDS polyacrylamide gel electrophoresis analysis**: SDS polyacrylamide gel electrophoresis (SDS‐PAGE) was carried out using an XCell SureLock™ Mini‐Cell Electrophoresis System from ThermoFisher Scientific (Invitrogen™ NuPAGE™ Novex™ Bis‐Tris gel, NuPAGE™ MES SDS running buffer). Protein molecular weights were approximated by comparison to a protein marker (Perfect Protein™ Markers, 15–150 kDa from Novagen). Briefly, a 10 μL aliquot of the reaction mixture was desalted with a Zeba™ Spin desalting column (ThermoFisher Scientific) and 5 μL of this solution was transferred to a 0.5 mL Eppendorf tube. NuPAGE™ LDS sample buffer (4×, 2.5 μL), NuPAGE™ reducing agent (10×, 1 μL), and H_2_O (1.5 μL) were added. The solution was denatured at 70 °C for 10 min, loaded onto a 4–12 % polyacrylamide NuPAGE™ Novex™ Bis‐Tris gel, and then subjected to electrophoresis (200 V, 35 min) with 1×NuPAGE™ MES SDS running buffer+NuPAGE™ antioxidant. Gels were visualized by Coomassie staining (Instant Blue from Expedeon).

**Materials**: 2‐(*tert*‐Butoxycarbonylamino)ethyl bromide **6**,^[17]^ sodium 2,3,4,6‐tetra‐*O*‐acetyl‐1‐thio‐β‐D‐galactopyranose **8**,^[34]^ sodium 1‐thio‐β‐D‐galactopyranose **9**,^[35]^ trifluoromethanesulfonyl azide (TfN_3_),^[36]^ tris‐triazolyl amine ligand tris[(1‐ethylacetate‐*1H*‐1,2,3‐triazol‐4‐yl) methyl]amine,^[37]^ and SBL‐Dha156 (**13**)^[24]^ were prepared as previously described. Qβ‐Hag16 (**14**),^[23]^ *S*sβG‐Aha43 (**15**),^[28]^ Np276‐Aha61 (**16**),^[28]^ and S*s*βG‐Hpg1‐Hpg43/‐Hpg43 (**17 a**/**b**)^[29]^ were cloned and expressed as previously described. All other reagents were used as received from commercial suppliers.

### Chemical synthesis

**Methyl 3,4,5‐tris[2‐(*tert*‐butoxycarbonylamino)ethoxy]benzoate (4)**:[^17^, ^38^] A mixture of methyl 3,4,5‐trihydroxybenzoate **5** (2 g, 10.9 mmol), 2‐(*tert*‐butoxycarbonylamino)ethyl bromide **6** (9.7 g, 43.4 mmol), dry potassium carbonate (6.8 g, 48.9 mmol), and TBAI (0.41 g, 1.1 mmol) in dry DMF (41 mL) was stirred at 80 °C under an atmosphere of argon for 6 days. The reaction mixture was cooled to room temperature, filtered through a short path of Celite® 545, and the solvent was evaporated. The residue was dissolved in EtOAc and washed with water, saturated aqueous NaHCO_3_, and brine. The combined organic layers were dried over MgSO_4_, filtered, and concentrated under reduced pressure. The residue was purified by column chromatography (from 1 : 9 to 1 : 1 EtOAc/petrol) to afford **4** as a white solid (4.9 g, 75 %). *R_f_* (1 : 1 EtOAc/hexane): 0.33. ^1^H NMR (400 MHz, CDCl_3_,): δ 7.30 (s, 2H, Ar), 5.82 (bs, 1H, NH), 5.39 (bs, 2H, NH), 4.12 (m, 6H, OCH_2_), 3.89 (s, 3H, OCH_3_), 3.58 (m, 4H, CH_2_NH), 3.42 (m, 2H, CH_2_NH), 1.45 (s, 27H, CH_3_, Boc). ^13^C NMR (100.6 MHz, CDCl_3_): δ 166.0 (C=O, CO_2_Me), 155.7, 155.6 (C=O, Boc), 152.0 (C‐3,5), 140.8 (C‐4), 125.5 (C‐1), 108.1 (C‐2,6), 79.5, 79.3 (C, Boc), 72.4, 68.4 (OCH_2_), 52.1 (OCH_3_), 40.4, 39.9 (CH_2_NH), 28.2 (CH_3_, Boc). HRMS (TOF ES^+^) *m/z*: [M+Na]^+^ Calcd for C_29_H_47_N_3_NaO_11_^+^, 636.3103; found, 636.3104.

**Methyl 3,4,5‐tris[2‐(2‐chloroacetamido)ethoxy]benzoate (7)**: **4** (1.81 g, 2.946 mmol) was dissolved in dry CH_2_Cl_2_ (5.5 mL) and 4 M HCl in dioxane (18 mL) was added at room temperature under an atmosphere of argon. The reaction mixture was stirred at the same temperature for 2 h. After complete conversion, the solvent was evaporated and dried under high vacuum to afford methyl 3,4,5‐tris(2‐aminoethoxy)benzoate trihydrochloride as a white solid (1.23 g, 99 %). Used in the next step without further purification. A mixture of this intermediate and NaHCO_3_ (1.48 g, 17.676 mmol) in 2 : 1 Et_2_O/H_2_O (8.8 mL) was cooled to 0 °C (ice/water) and chloroacetyl chloride (771 μL, 9.721 mmol) was slowly added over a period of 1 h. After complete addition, the mixture was allowed to warm to room temperature. After 7 days stirring at the same temperature, the reaction mixture was filtered. The precipitate was washed with water, 2 N aqueous HCl, water, and finally with Et_2_O. The crude product was recrystallized from 1 : 1 ethanol/water and dried under reduced pressure to afford **7** as a white solid (1.53 g, 97 % over two steps). *R_f_* (EtOAc): 0.23. mp (EtOH/water 1 : 1): 158–160 °C. ^1^H NMR (400 MHz, DMSO‐*d*_6_): δ 8.43 (bs, 2H, NH), 8.25 (bs, 1H, NH), 7.24 (s, 2H, Ar), 4.10–4.01 (m, 12H, CH_2_Cl, OCH_2_), 3.83 (s, 3H, OCH_3_), 3.51–3.36 (m, 6H, CH_2_NH). ^13^C NMR (100.6 MHz, DMSO‐*d*_6_): δ 166.4, 166.2 (C=O, C(O)CH_2_Cl), 165.7 (C=O, CO_2_Me), 151.9 (C‐3,5), 141.2 (C‐4), 124.8 (C‐1), 107.9 (C‐2,6), 71.0, 67.2 (OCH_2_), 52.3 (OCH_3_), 42.6 (CH_2_Cl), 39.5, 38.8 (CH_2_NH). FT‐IR (KBr, *ν*_max_): 3258, 2978, 2874, 1721, 1644. HRMS (TOF ES^+^) *m/z*: [M+Na]^+^ Calcd for C_20_H_26_Cl_3_N_3_NaO_8_^+^, 564.0678; found, 564.0678.

**Methyl 3,4,5‐tris{2‐[2‐(2,3,4,6‐tetra‐*O*‐acetyl‐1‐thio‐β‐D‐galactopyranosyl)acetamido]ethoxy}benzoic acid (10)**: Sodium 2,3,4,6‐tetra‐*O*‐acetyl‐1‐thio‐β‐D‐galactopyranoside **8** (47 mg, 0.122 mmol) was added to a dispersion of **7** (20 mg, 0.037 mmol) in dry DMF (962 μL) at room temperature with an argon stream bubbling through the solution. The reaction mixture was stirred at the same temperature for 3 days. The solvent was evaporated and the crude was purified by column chromatography (from 1 : 1 EtOAc/petrol to EtOAc) to afford **10** as a colourless syrup (18.8 mg, 33 %). *R_f_* (7 : 3 : 1 *i*PrOH/H_2_O/NH_4_OH): 0.88. [α]_D_^20^+110.0 (*c* 0.01, CHCl_3_). ^1^H NMR (500 MHz, CDCl_3_,): δ 7.47 (bs, 1H, NH), 7.32 (bs, 2H, NH), 7.29 (s, 2H, Ar), 5.41 (d, *J*_3’,4’_=3 Hz, 3H, H‐4’), 5.20 (appt, *J*_1’,2’_=10 Hz, *J*_2’,3’_=9.5 Hz, 3H, H‐2’), 5.10 (dd, *J*_2’,3’_=9.5 Hz, *J*_3’,4’_=3.5 Hz, 3H, H‐3’), 4.74 (d, *J*_1’,2’_=10 Hz, 1H, H‐1’), 4.70 (d, *J*_1’,2’_=10 Hz, 2H, H‐1’), 4.15–4.07 (m, 12H, H‐6’ab, OCH_2_), 3.97 (m, 3H, H‐5’), 3.90 (s, 3H, OCH_3_), 3.73–3.52 (m, 6H, CH_2_NH), 3.37–3.29 (m, 6H, CH_2_S), 2.14–1.98 (bs, 36H, CH_3_). ^13^C NMR (125.8 MHz, CDCl_3_,): δ 171.2–169.2 (C=O, Ac, C(O)CH_2_S), 166.1 (C=O, CO_2_Me), 152.0 (C‐3,5), 141.1 (C‐4), 125.8 (C‐1), 108.3 (C‐2,6), 83.5 (C‐1’), 74.6 (C‐5’), 71.5 (C‐3’, OCH_2_), 68.0 (OCH_2_), 67.18, 67.15 (C‐2’,4’), 61.3, 60.4 (C‐6’), 52.4 (OCH_3_), 40.3, 39.2 (CH_2_NH), 33.6, 29.7 (CH_2_S), 20.7–20.6 (CH_3_, Ac). FT‐IR (NaCl, *ν*_max_): 3383, 3064, 2958, 2935, 2854, 1749, 1668, 1558. Anal. Calcd for C_62_H_83_N_3_O_35_S_3_: C, 48.78; H, 5.48; N, 2.75; S, 6.30. Found: C, 48.78; H, 5.49; N, 2.74; S, 6.31. MS (TOF ES^+^) *m/z* (%): [M+Na]^+^ Calcd isotope ratios for C_62_H_83_N_3_NaO_35_S_3_^+^, 1548.39 (100), 1549.39 (67), 1550.39 (22), 1550.38 (14), 1551.39 (5), 1552.39 (3); found, 1548.38 (100), 1549.38 (68), 1550.38 (42), 1551.38 (19), 1552.38 (7).

**3,4,5‐Tris{2‐[2‐(1‐thio‐β‐D‐galactopyranosyl)acetamido]ethoxy}benzoic acid (11)**: Sodium 1‐thio‐β‐D‐galactopyranose **9** (10 mg, 0.046 mmol) was added to a dispersion of **7** (7.5 mg, 0.014 mmol) in dry DMF (360 μL) at room temperature with an argon stream bubbling through the solution. The reaction mixture was stirred at the same temperature for 24 h. The solvent was then evaporated and the crude treated with 1 N aqueous NaOH (322 μL) in EtOH (3.2 mL) at room temperature for 22 h. After neutralization with Dowex (H^+^ 50WX8‐200), the ion exchanger was filtered off and washed with water. The crude was purified by gel permeation chromatography (Bio‐gel® P‐2, H_2_O) followed by lyophilization to afford **11** as a white solid (264 mg, 74 % over two steps). *R_f_* (7 : 3 : 1 *i*PrOH/H_2_O/NH_4_OH): 0.05. mp 111–113 °C. [α]_D_^20^ −52.5 (*c* 0.02, H_2_O). ^1^H NMR (500 MHz, CD_3_OD): δ 7.27 (s, 2H, Ar), 4.43 (d, *J*_1’,2’_=9.5 Hz, 1H, H‐1’), 4.40 (d, *J*_1’,2’_=9.5 Hz, 2H, H‐1’), 4.23 (m, 6H, OCH_2_), 3.92–3.86 (m, 3H, H‐4’), 3.73–3.39 (m, 27H, H‐2’,3’,5’,6’ab, CH_2_NH, CH_2_S). ^13^C NMR (125.8 MHz, CD_3_OD): δ 174.5 (C=O, CO_2_H), 173.6, 173.5 (C=O, C(O)CH_2_S), 152.5 (C‐3,5), 139.7 (C‐4), 133.4 (C‐1), 108.9 (C‐2,6), 86.7, 86.3 (C‐1’), 80.2 (C‐5’), 75.0 (C‐3’), 70.80, 69.9, 68.4 (C‐2’,4’, OCH_2_), 62.2 (C‐6’), 41.4, 40.5 (CH_2_NH), 35.6, 34.2 (CH_2_S). FT‐IR (KBr, *ν*_max_): 3735, 3629, 3005, 2990, 1653, 1559. Anal. Calcd for C_37_H_57_N_3_O_23_S_3_: C, 44.09; H, 5.70; N, 4.17; S, 9.54. Found: C, 44.11; H, 5.71; N, 4.19; S, 9.52. MS (TOF ES^+^) *m/z* (%): [M+Na]^+^ Calcd isotope ratios for C_37_H_57_N_3_NaO_23_S_3_^+^, 1030.24 (100), 1031.25 (40), 1032.24 (14), 1032.25 (8), 1033.24 (5), 1034.25 (1); found, 1030.24 (100), 1031.24 (22), 1032.24 (9), 1033.24 (1), 1034.24 (0.2).

**3,4,5‐Tris{2‐[2‐(1‐thio‐β‐D‐galactopyranosyl)acetamido]ethoxy}‐*N*‐[2‐(thioethyl)]benzamide (1)**: A mixture of **11** (15 mg, 0.015 mmol), cystamine (27 mg, 0.018 mmol), HATU (7 mg, 0.018 mmol), and DIPEA (18 μL, 0.104 mmol) in dry DMF (0.5 mL) was stirred at 50 °C under an atmosphere of argon for 3 days. The solvent was evaporated and the crude was diluted with degassed water (0.3 mL). PBu_3_ (3 μL) was then added and the mixture was stirred at room temperature for 2 h. The crude was purified by gel permeation chromatography (Bio‐gel® P‐2, degassed H_2_O) followed by lyophilization to afford **1** as a white solid (11 mg, 69 % over two steps). *R_f_* (7 : 3 : 1 *i*PrOH/H_2_O/NH_4_OH): 0.36. [α]_D_^20^ +7.7 (*c* 0.02, H_2_O). ^1^H NMR (500 MHz, D_2_O): δ 7.11 (s, 2H, Ar), 4.38 (d, *J*_1’,2’_=9.7 Hz, 1H, H‐1’), 4.36 (d, *J*_1’,2’_=9.7 Hz, 2H, H‐1’), 4.21–4.15 (m, 6H, OCH_2_), 3.90–3.83 (m, 3H, H‐4’), 3.71–3.35 (m, 27H, H‐2’,3’,5’,6’ab, CH_2_NH, CH_2_S), 3.41–2.67 (m, 4H, CH_2_CH_2_). ^13^C NMR (125.8 MHz, D_2_O): δ 172.5, 172.3 (C=O, C(O)CH_2_S), 169.3 (C=O, C(O)NHCH_2_CH_2_), 151.6 (C‐3,5), 138.9 (C‐4), 129.6 (C‐1), 106.0 (C‐2,6), 85.4, 85.1 (C‐1’), 79.0 (C‐5’), 73.8 (C‐3’), 71.4 (OCH_2_), 69.5 (C‐2’), 68.7 (C‐4’), 67.3 (OCH_2_), 61.0 (C‐6’), 40.2, 39.3, 37.4, 33.1, 23.2 (CH_2_NH, CH_2_CH_2_, CH_2_S). FT‐IR (KBr, *ν*_max_): 3355, 2870, 2831, 1619, 1527, 1275, 1136, 775, 573. HRMS (TOF ES^+^) *m/z*: [M+Na]^+^ Calcd for C_39_H_62_N_4_NaO_22_S_4_^+^, 1089.2631; found, 1089.2624.

**3,4,5‐Tris{2‐[2‐(1‐thio‐β‐D‐galactopyranosyl)acetamido]ethoxy}‐*N*‐(prop‐2‐ynyl)benzamide (2)**: A mixture of **11** (15 mg, 0.015 mmol), propargylamine hydrochloride (2 mg, 0.018 mmol), HATU (7.4 mg, 0.019 mmol), and DIPEA (9 μL, 0.052 mmol) in dry DMF (420 μL) was stirred at 45 °C under an atmosphere of argon for 27 h. The solvent was evaporated and the crude was purified by gel permeation chromatography (Bio‐gel® P‐2, H_2_O) followed by lyophilization to afford **11** as a white solid (11.7 mg, 75 %). *R_f_* (7 : 3 : 1 *i*PrOH/H_2_O/NH_4_OH): 0.23. mp 98–100 °C. [α]_D_^20^: +24.0 (*c* 0.03, H_2_O). ^1^H NMR (500 MHz, CD_3_OD): δ 7.15 (s, 2H, Ar), 4.45 (d, *J*_1’,2’_=9.5 Hz, 1H, H‐1’), 4.40 (d, *J*_1’,2’_=9.5 Hz, 2H, H‐1’), 4.24 (m, 6H, OCH_2_), 4.14 (m, 2H, CH_2_C≡), 3.94–3.87 (m, 3H, H‐4’), 3.75–3.20 (m, 28H, H‐2’,3’,5’,6’ab, ≡CH, CH_2_NH, CH_2_S). ^13^C NMR (125.8 MHz, CD_3_OD): δ 173.7, 173.5 (C=O, C(O)CH_2_S), 170.3 (C=O, C(O)NHCH_2_CH_2_), 153.0 (C‐3,5), 140.4 (C‐4), 130.5 (C‐1), 107.3 (C‐2,6), 86.6, 86.3 (C‐1’), 80.7 (C≡), 80.2 (C‐5’), 75.0 (C‐3’), 73.0 (≡CH), 70.7, 69.9, 68.5 (C‐2’,4’, OCH_2_), 62.2 (C‐6’), 40.4, 39.9 (CH_2_NH), 34.3, 34.2 (CH_2_S), 30.5 (CH_2_). FT‐IR (KBr, *ν*_max_): 3583, 3406, 2995, 2122, 1646, 1636, 1558. Anal. Calcd for C_40_H_60_N_4_O_22_S_3_: C, 45.97; H, 5.79; N, 5.36; S, 9.20. Found: C, 45.99; H, 5.77; N, 5.38; S, 9.18. MS (TOF ES^+^) *m/z* (%): [M+Na]^+^ Calcd isotope ratios for C_40_H_60_N_4_NaO_22_S_3_^+^, 1067.28 (100), 1068.28 (43), 1069.28 (30), 1069.27 (14), 1070.27 (6); found 1067.27 (100), 1068.28 (23), 1069.27 (8), 1070.28 (1).

**3,4,5‐Tris{2‐[2‐(1‐thio‐β‐D‐galactopyranosyl)acetamido]ethoxy}‐*N*‐[2‐(*tert*‐butoxycarbonylamino)ethyl]benzamide (12)**: A mixture of **11** (15 mg, 0.015 mmol), *N*‐Boc‐ethylenediamine (3 μL, 0.018 mmol), HATU (7.4 mg, 0.019 mmol), and DIPEA (6 μL, 0.037 mmol) in dry DMF (417 μL) was stirred at 45 °C under an atmosphere of argon for 27 h. The solvent was evaporated and the crude was purified by gel permeation chromatography (Bio‐gel® P‐2, H_2_O) followed by lyophilization to afford **12** as a white solid (11.5 mg, 67 %). *R_f_* (7 : 3 : 1 *i*PrOH/H_2_O/NH_4_OH): 0.36. mp 104–106 °C. [α]_D_^20^: +7.7 (*c* 0.02, H_2_O). ^1^H NMR (500 MHz, CD_3_OD): δ 7.13 (s, 2H, Ar), 4.46 (d, *J*_1’,2’_=9.5 Hz, 1H, H‐1’), 4.42 (d, *J*_1’,2’_=9.5 Hz, 2H, H‐1’), 4.24–4.20 (m, 6H, OCH_2_), 3.94–3.88 (m, 3H, H‐4’), 3.73–3.40 (m, 31H, H‐2’,3’,5’,6’ab, CH_2_NH, CH_2_S, CH_2_CH_2_), 1.34 (s, 9H, CH_3_, Boc). ^13^C NMR (125.8 MHz, CD_3_OD): δ 173.7, 173.6 (C=O, C(O)CH_2_S), 169.1 (C=O, C(O)NHCH_2_CH_2_), 156.8 (C=O, Boc, C‐3,5), 146.4 (C‐4), 125.8 (C‐1), 107.3 (C‐2,6), 86.6, 86.3 (C‐1’), 80.2 (C‐5’, C, Boc), 75.0 (C‐3’), 70.7, 69.9, 68.5 (C‐2’,4’, OCH_2_), 62.2 (C‐6’), 41.3, 40.4, 34.2 (CH_2_NH, CH_2_CH_2_, CH_2_S), 28.7 (CH_3_, Boc). FT‐IR (KBr, *ν*_max_): 3323, 3005, 2976, 2928, 1683, 1651, 1557. Anal. Calcd for C_44_H_71_N_5_O_24_S_3_: C, 45.95; H, 6.22; N, 6.09; S, 8.36. Found: C, 45.97; H, 6.22; N, 6.11; S, 8.38. MS (TOF ES^+^) *m/z* (%): [M+Na]^+^ Calcd isotope ratios for C_44_H_71_N_5_NaO_24_S_3_^+^, 1172.35 (100), 1173.36 (48), 1174.35 (14), 1174.36 (11), 1175.36 (2), 1176.36 (2); found, 1172.31 (100), 1173.32 (49), 1174.31 (28), 1175.32 (10), 1176.31 (4).

**3,4,5‐Tris{2‐[2‐(1‐thio‐β‐D‐galactopyranosyl)acetamido]ethoxy}‐*N*‐(2‐azidoethyl)benzamide (3)**: **12** (4 mg, 3.5 μmol) was dissolved in a freshly prepared solution of 1 : 2 Me_2_S/TFA (100 μL) at 0 °C (ice/water) and stirred for 3 h. After complete conversion, the solvent was evaporated and dried under high vacuum. The crude was dissolved in MeOH (45 μL) and 10 mol% CuSO_4_ (0.1 mg, 0.4 μmol) and DMAP (0.8 mg, 7 μmol) were added at 0 °C (ice/water). Freshly prepared *ca*. 0.4 M TfN_3_ in CH_2_Cl_2_ (200 μL) was added at the same temperature. After complete addition, the mixture was allowed to warm to room temperature. After 19 h stirring at the same temperature, the solvent was evaporated and the crude was purified by gel permeation chromatography (Bio‐gel® P‐2, H_2_O) followed by lyophilization to afford **3** as a yellowish foam (3.5 mg, 95 % over two steps). *R_f_* (7 : 3 : 1 *i*PrOH/H_2_O/NH_4_OH): 0.37. mp 106–109 °C. [α]_D_^20^: −26.0 (*c* 0.02, H_2_O). ^1^H NMR (500 MHz, CD_3_OD): δ 7.15 (s, 2H, Ar), 4.45 (d, *J*_1’,2’_=9.5 Hz, 1H, H‐1’), 4.41 (d, *J*_1’,2’_=9.5 Hz, 2H, H‐1’), 4.23 (m, 6H, OCH_2_), 4.08–3.87 (m, 3H, H‐4’), 3.72–3.39 (m, 31H, H‐2’,3’,5’,6’ab, CH_2_NH, CH_2_S, CH_2_CH_2_). ^13^C NMR (125.8 MHz, CD_3_OD): δ 173.7 (C=O, C(O)CH_2_S), 153.0 (C‐3,5), 126.2 (C‐1), 107.2 (C‐2,6), 86.6, 86.3 (C‐1’), 80.2 (C‐5’), 75.0 (C‐3’), 70.7, 69.9, 69.6, 68.5 (C‐2’,4’, OCH_2_), 62.2 (C‐6’), 51.2 (CH_2_N_3_), 40.7, 40.4, 34.3, 34.2 (CH_2_NH, CH_2_, CH_2_S). FT‐IR (KBr, *ν*_max_): 3418, 3006, 2986, 2937, 2110, 1681, 1651, 1456, 1167. Anal. Calcd for C_39_H_61_N_7_O_22_S_3_: C, 43.53; H, 5.71; N, 9.11; S, 8.94. Found: C, 43.49; H, 5.70; N, 9.10; S, 8.91. MS (TOF ES^+^) *m/z* (%): [M+Na]^+^ Calcd isotope ratios for C_39_H_61_N_7_NaO_22_S_3_^+^, 1098.29 (100), 1099.30 (42), 1100.29 (14), 1100.30 (9), 1101.29 (6), 1102.29 (1); found 1098.28 (100), 1099.29 (47), 1100.29 (27), 1101.29 (11), 1102.29 (4).

### Chemical protein modification

**Gal_3_‐G‐*S*‐156SBL (18)**: Gal_3_‐G‐SH **1** (1.0 mg, 0.937 μmol) was added to a solution of SBL‐Dha156 (**13**) (100 μL of 0.25 mg/mL, 0.937 nmol) in 50 mM sodium phosphate buffer (pH 8.0) and the resulting mixture vortexed for 30 seconds at room temperature. After 1.5 h of additional shaking, a 30 μL aliquot was analysed directly by LC–MS and complete conversion to Gal_3_‐G‐*S*‐156SBL **18** (calcd. 27748; found, 27749) was observed. Finally, the sample was flash frozen with liquid nitrogen and stored at −20 °C. *Note*: The modified glycodendriprotein retained inherent peptidase activity, as indicated by liberation of *p*‐nitroaniline upon treatment with the chromogenic peptide sucAAPF*p*NA.^[39]^

**Stability of Gal_3_‐G‐*S*‐156SBL (18) in human plasma**: A 10 μL aliquot of Gal_3_‐G‐*S*‐156SBL (**18**) (*ca*. 0.25 mg/mL) in 50 mM sodium phosphate buffer (pH 8.0) was transferred to a 0.5 mL Eppendorf tube. 0.5 μL of reconstituted human plasma (Sigma‐Aldrich) was added at room temperature and the resulting mixture vortexed for 30 seconds. After 24 h of additional shaking at 37 °C, the reaction was analysed directly by LC–MS and starting protein **18** (calculated mass, 27748; observed mass, 27752) was detected unaltered.

**Gal_3_‐G‐*S*‐16Qβ (19)**: Gal_3_‐G‐SH **1** (4.5 mg, 4.218 μmol) and Vazo44 (0.28 mg, 0.844 μmol) were added to a solution of Qβ‐Hag16 (**14**) (100 μL of 1.19 mg/mL, 8.437 nmol) in 250 mM ammonium acetate buffer (pH 4.0 or 6.0). The reaction mixture was placed in a cuvette and irradiated with a medium pressure 125 W Hg‐lamp with borosilicate filter at room temperature for up to 28 h. Small molecules were removed from the reaction mixture aliquot by loading the sample onto a PD10 desalting column (GE Healthcare) previously equilibrated with 10 column volumes 50 mM sodium phosphate buffer (pH 8.0) and eluting with 1 mL of the same buffer. The collected sample was concentrated to 50 μL on a Vivaspin™ membrane concentrator (10 kDa molecular weight cut off). A virus‐like particle aliquot (20 μL) was mixed with 1 M DTT (Dithiothreitol) in H_2_O (10 μL) and incubated at 60 °C for 5 min to allow the protein to denature to monomer prior to analysis by LC–MS (*m*/*z* for monomer of Gal_3_‐G‐*S*‐16Qβ **19**: calcd. 15173; found, 15173). Finally, the sample was flash frozen with liquid nitrogen and stored at −20 °C.

**Gal_3_‐G‐*triazole*‐43*S*sβG (20)**: Gal_3_‐G‐propargyl **2** (1.95 mg, 1.87 μmol) was dissolved in 50 mM sodium phosphate buffer (50 μL, pH 8.2). A freshly prepared solution of copper(I) bromide (99.999 %) in acetonitrile (163 μL, 10 mg/mL) was premixed with an acetonitrile solution of tris‐triazolyl amine ligand tris[(1‐ethylacetate‐*1H*‐1,2,3‐triazol‐4‐yl) methyl]amine (63 μL, 127 mg/mL). The preformed Cu‐complex solution (25 μL) was added to the above solution and mixed thoroughly. *S*sβG‐Aha43 (**15**) (100 μL, 0.5 mg/mL) was added to the mixture and the reaction was agitated on a rotator at room temperature for 1 h. 0.2 mL of High Affinity Ni‐Charged resin was then added to the mixture and the reaction was agitated on a rotator at 4 °C for 1 h. The sample was then placed in a syringe and eluted with 5‐column volume of buffer A (low imidazole concentration; 20 mM Tris HCl, 500 mM NaCl, 5 mM imidazole, pH 7.8) and 5‐column volume of buffer B (high imidazole concentration; 20 mM Tris HCl, 500 mM NaCl, 15 mM imidazole, pH 7.8). The eluted buffer B was concentrated on a Vivaspin™ membrane concentrator (10 kDa molecular weight cut off) and washed with 50 mM sodium phosphate buffer (3× 200 μL, pH 7.0). The solution was concentrated to 100 μL and the product was characterized by LC–MS (calcd. 58276; found, 58285). Finally, the sample was flash frozen with liquid nitrogen and stored at −20 °C. *Note*: The modified glycodendriprotein retained galactosidase activity as evidenced by X‐Gal stain.^[40]^

**Stability of Gal_3_‐G‐*triazole*‐43*S*sβG (20) in human plasma**: A 10‐μL aliquot of Gal_3_‐G‐*triazole*‐43*S*sβG (**20**) (*ca*. 0.5 mg/mL) in 50 mM sodium phosphate buffer (pH 7.0) was transferred to a 0.5 mL Eppendorf tube. Reconstituted human plasma (0.5 μL; Sigma‐Aldrich) was added at room temperature and the resulting mixture vortexed for 30 seconds. After 24 h of additional shaking at 37 °C, the reaction was analysed directly by LC–MS and starting protein **20** (calculated mass, 58276; observed mass, 58277) was detected unaltered.

**Gal_3_‐G‐*triazole*‐61Np276 (21)**: Gal_3_‐G‐propargyl **2** (1.86 mg, 1.78 μmol) was dissolved in 50 mM sodium phosphate buffer (400 μL, pH 8.2). A freshly prepared solution of copper(I) bromide (99.999 %) in acetonitrile (162 μL, 10 mg/mL) was premixed with an acetonitrile solution of tris‐triazolyl amine ligand tris[(1‐ethylacetate‐*1H*‐1,2,3‐triazol‐4‐yl) methyl]amine (62 μL, 127 mg/mL). The preformed Cu‐complex solution (45 μL) was added to the above solution and mixed thoroughly. Np276‐Aha61 (**16**) (100 μL, 2 mg/mL) was added to the mixture and the reaction was agitated on a rotator at room temperature for 1 h. The protein solution was dialyzed against 50 mM sodium phosphate buffer (pH 7.0) with EDTA (10 mM) and DTT (5 mM) at 4 °C for 4 h and against 50 mM sodium phosphate buffer (pH 7.0) at 4 °C for 10 h. The product was characterized by LC–MS (calcd. 21796; found, 21794). Finally, the sample was flash frozen with liquid nitrogen and stored at −20 °C.

**Gal_3_‐G‐*triazole*‐1*S*sβG (22)**: Gal_3_‐G‐N_3_ **3** (1.18 mg, 1.10 μmol) was dissolved in 50 mM sodium phosphate buffer (50 μL, pH 8.2). A freshly prepared solution of copper(I) bromide (99.999 %) in acetonitrile (163 μL, 10 mg/mL) was premixed with an acetonitrile solution of tris‐triazolyl amine ligand tris[(1‐ethylacetate‐1*H*‐1,2,3‐triazol‐4‐yl) methyl]amine (63 μL, 127 mg/mL). The preformed Cu‐complex solution (25 μL) was added to the above solution and mixed thoroughly. *S*sβG‐Hpg1‐Hpg43/‐Hpg43 (**17 a**/**b**) solution (100 μL, 0.6 mg/mL) was added to the mixture and the reaction was agitated on a rotator for 1 h at room temperature. 0.2 mL of High Affinity Ni‐Charged resin was then added to the mixture and the reaction was agitated on a rotator at 4 °C for 1 h. The sample was then placed in a syringe and eluted with 5‐column volume of buffer A (low imidazole concentration; 20 mM Tris HCl, 500 mM NaCl, 5 mM imidazole, pH 7.8) and 5‐column volume of buffer B (high imidazole concentration; 20 mM Tris HCl, 500 mM NaCl, 15 mM imidazole, pH 7.8). The eluted buffer B was concentrated on a Vivaspin™ membrane concentrator (10 kDa molecular weight cut off) and washed with 50 mM sodium phosphate buffer (3×200 μL, pH 7.0). The solution was concentrated to 100 μL the product was characterized by LC–MS (calcd. 58398; found, 58404). Finally, the sample was flash frozen with liquid nitrogen and stored at −20 °C. *Note*: The modified glycoproteins retained galactosidase activity as evidenced by X‐Gal stain.^[40]^ In addition, regioselective monomodification only at position 1 was observed for dialkynic protein *S*sβG‐Hpg1‐Hpg43 (**17 a**) together with residual, post‐translationally modified *S*sβG‐Hpg43 (**17 b**) resulting from *N*‐terminal Hpg excision in **15 a** (calcd. 57214; found, 57218). This result is in agreement with results previously reported.^[29]^ For a detailed discussion on reactive accessibility of sites 1 and 43 on *S*sβG‐Hpg1‐Hpg43 (**17 a**), see work by van Kasteren *et al*.^[33]^ and corresponding Supporting Information.

## Conflict of interest

The authors declare no conflict of interest.

1

## Supporting information

As a service to our authors and readers, this journal provides supporting information supplied by the authors. Such materials are peer reviewed and may be re‐organized for online delivery, but are not copy‐edited or typeset. Technical support issues arising from supporting information (other than missing files) should be addressed to the authors.

Supporting Information

## Data Availability

The data that support the findings of this study are available in the supplementary material of this article.
