# Supplementary material for: Probing Site‐Selective Conjugation Chemistries for the Construction of Homogeneous Synthetic Glycodendriproteins
Source: Chembiochem. 2022 Apr 1;23(10):e202200020. doi: 10.1002/cbic.202200020 (PMC9322419; doi:10.1002/cbic.202200020)
Supplement: Supplementary file 1 — Supporting Information [file CBIC-23-0-s001.pdf]

ChemBioChem

Supporting Information

Probing Site-Selective Conjugation Chemistries for the Construction of Homogeneous Synthetic Glycodendriproteins

Isidro Cobo, M. Isabel Matheu, Sergio Castillón, Benjamin G. Davis,* and Omar Boutureira*

Table of Contents

1. Attempted Syntheses of Glycodendron Reagents and Optimization Reactions S2

2. ESI–MS Spectra S4

3. SDS-PAGE Gels S12

4. NMR Spectra S13

5. References S28

1. Attempted Syntheses of Glycodendron Reagents and Optimization Reactions

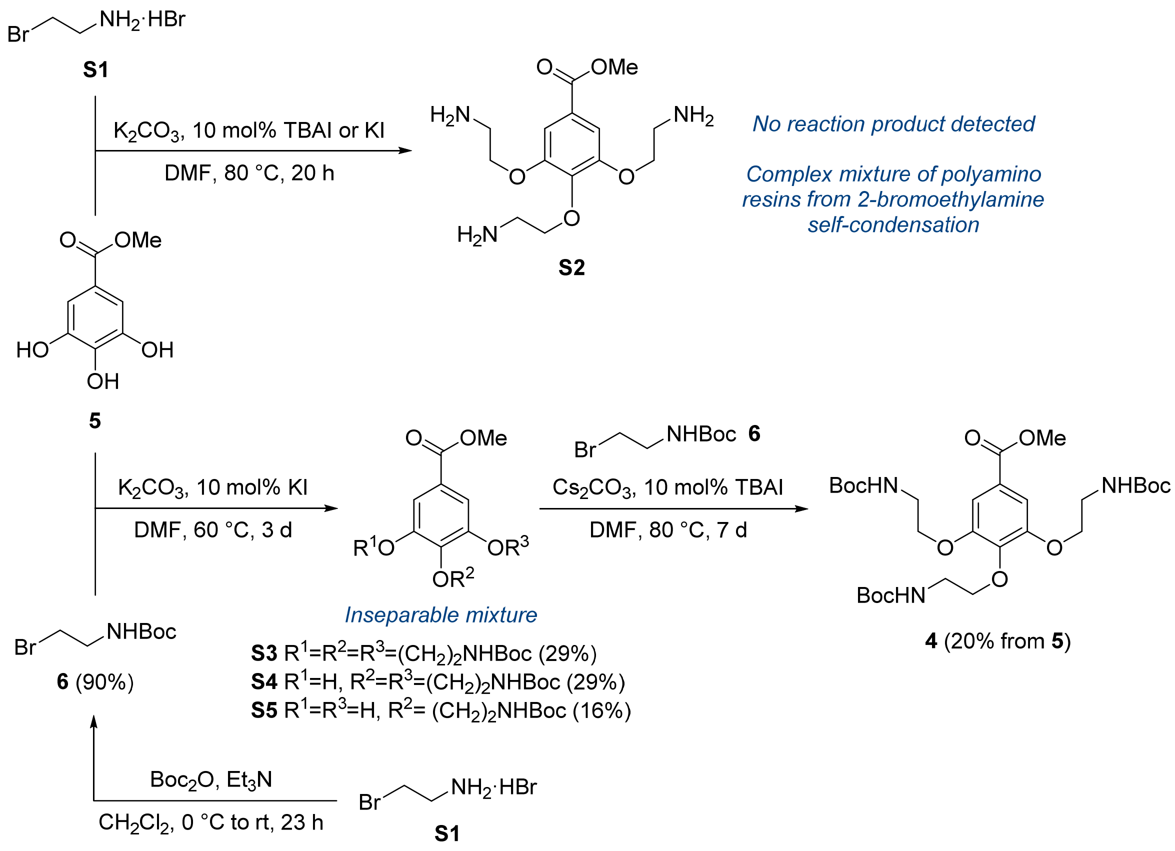

Scheme S1. Attempted synthesis of **S2** and optimization reactions for the preparation of **4**

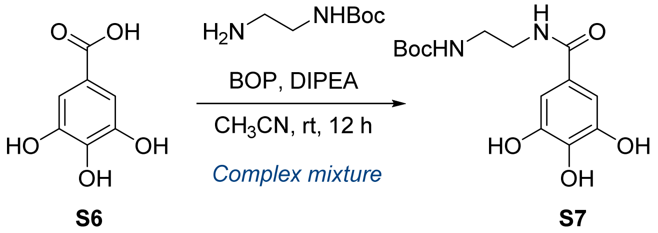

Scheme S2. Attempted synthesis of **S7** from **S6**

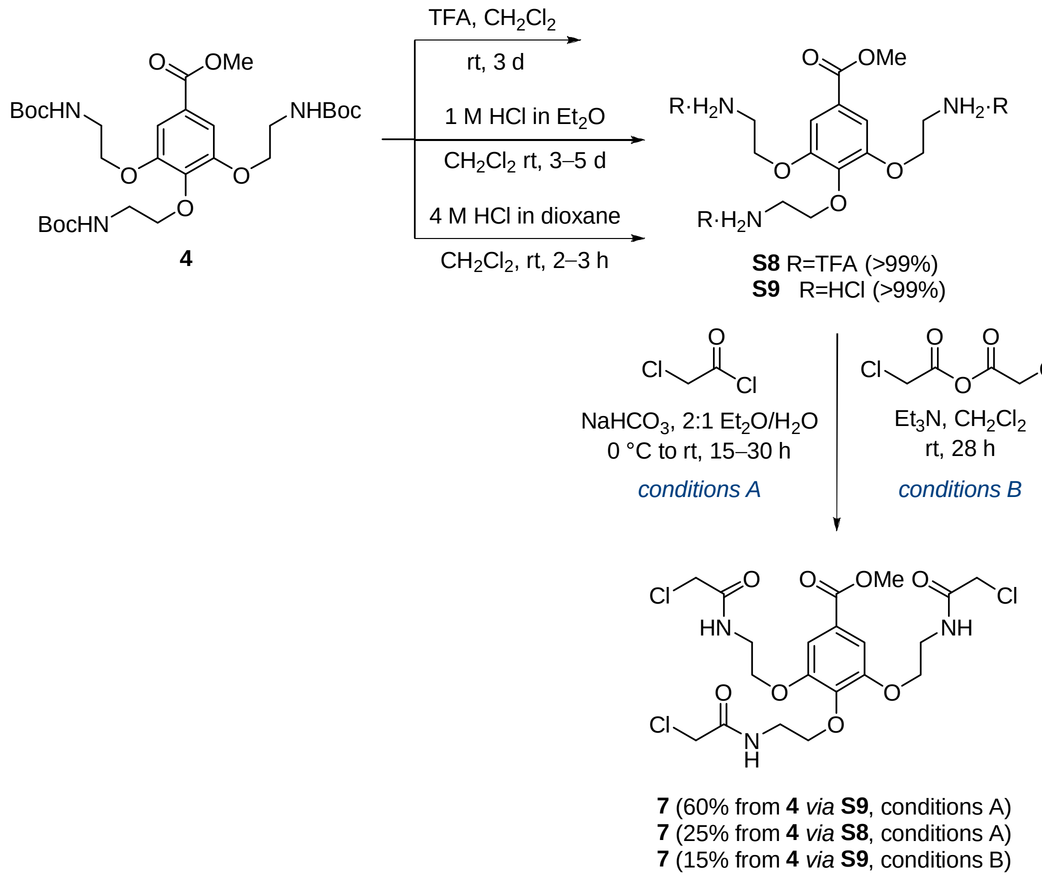

Scheme S3. Attempted deprotection of **4** and optimization reactions for the preparation of **7**

Table S1. Optimization of the synthesis of glycodendron **11**

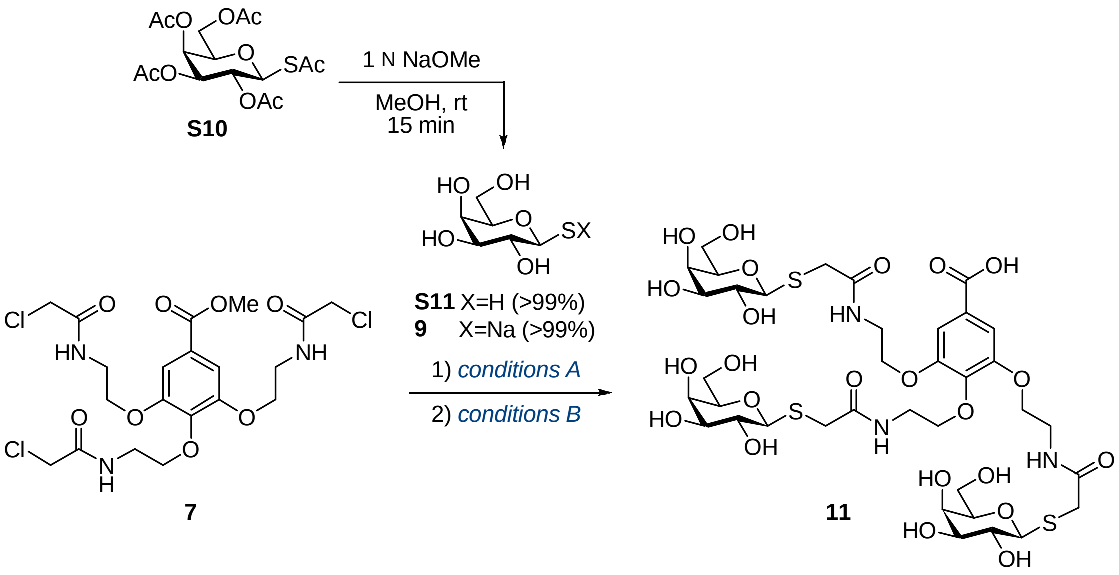

entry	sugar(X)	conditions A (v/v)	conditions B (v/v)	product (%)
1	βGalS(H)	1% Et_3N, (2:1) $DMSO/H_2O$, rt, 4 d	(1:2) 2 N $NaOH_{aq}$/EtOH rt, 38 h	mixture
2	βGalS(H)	1% DIPEA, (2:1) $DMSO/H_2O$, rt, 4 d	(1:2) 2 N $NaOH_{aq}$/EtOH rt, 8 h	mixture
3[a]	βGalS(Na)	DMF, rt, 1–4 d	(1:10) 1 N $NaOH_{aq}$/EtOH rt, 22–24 h	**11** (59–74)
4[b]	βGalS(Na)	DMF, rt, 24 h	*t*-BuOK, H_2O, Et_2O rt, 2 h	mixture
5	βGalS(Na)	DMSO, rt, 24 h	(1:10) 1 N $NaOH_{aq}$/EtOH rt, 23 h	**11** (50)
6[c]	βGalS(Na)	DMSO, rt, 24 h	0.54 M EtSLi in HMPA DMSO, rt, 20 h	mixture
7	βGalS(Na)	(2:1) DMF/H_2O, rt, 2 d	(1:10) 1 N $NaOH_{aq}$/EtOH rt, 45 h	**11** (74)

[a]See reference 1. [b]See references 2,3. [c]See reference 4.

2. ESI–MS Spectra

Subtilisin from *Bacillus lentus* (SBL)[5–11]

Sequence of SBL-C156Dha mutant (modified residue highlighted)

AQSVPWGISRVQAPAAHNRGLTGSGVKVAVLDTGISTHPDLNIRGGASFVPGEPSTQDGN
GHGTHVAGTIAALNNSIGVLGVAPSAELYAVKVLGASGSGSVSSIAQGLEWAGNNGMHV
ANLSLGSPSPSATLEQAVNSATSRGVLVVAASGNDhaGAGSISYPARYANAMAVGATDQN
NNRASFSQYGAGLDIVAPGVNVQSTYPGSTYASLNGTSMATPHVAGAALVKQKNPSWS
NVQIRNHLKNTATSLGSTNLYGSGLVNAEAATR

Calculated average isotopic mass = 26680.48 Da (*N*-terminal Met cleaved)

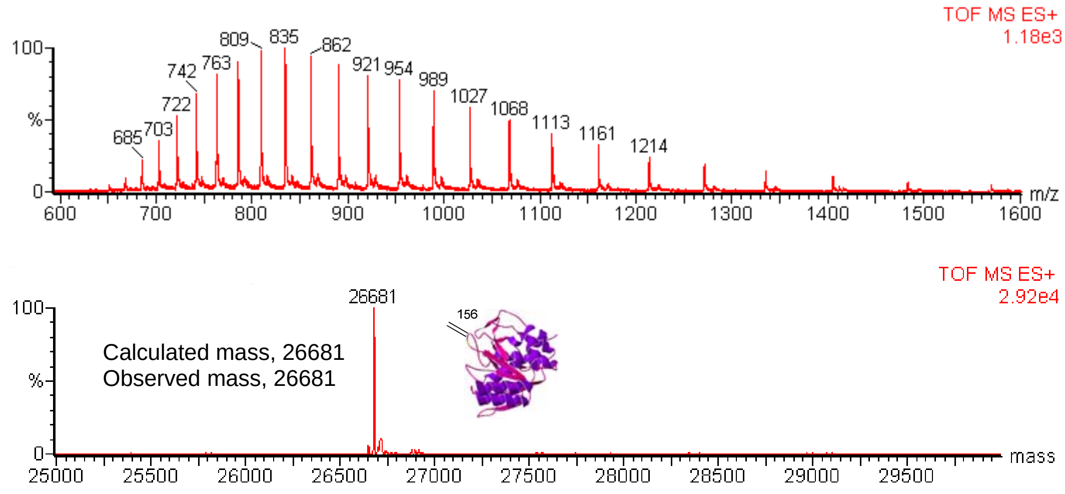

Figure S1. ESI–MS spectrum of SBL-Dha156 (**13**)

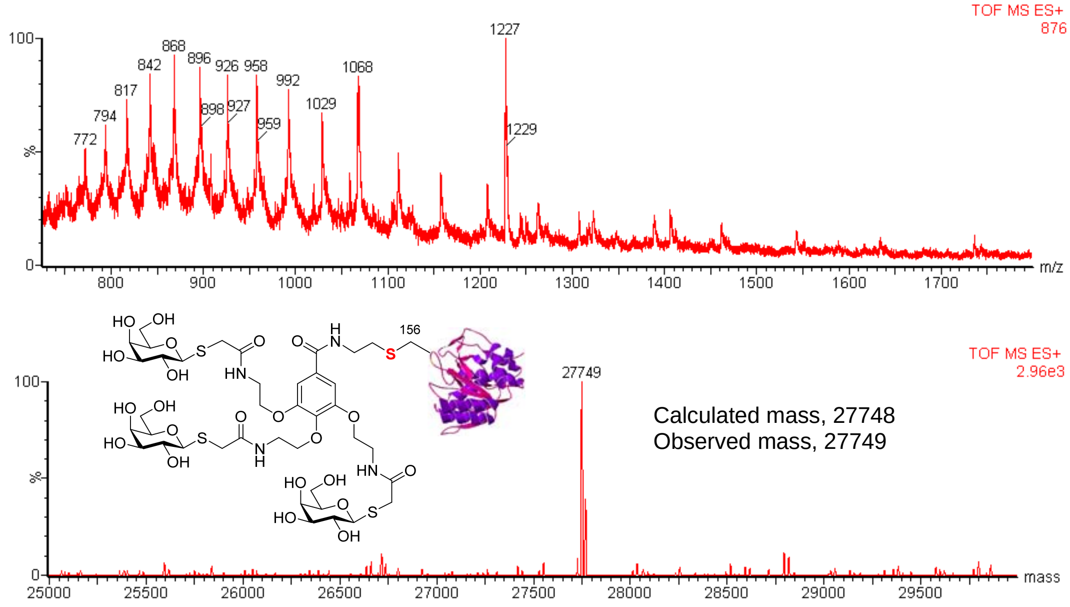

Figure S2. ESI–MS spectrum of GGal-*S*-156SBL (**18**)

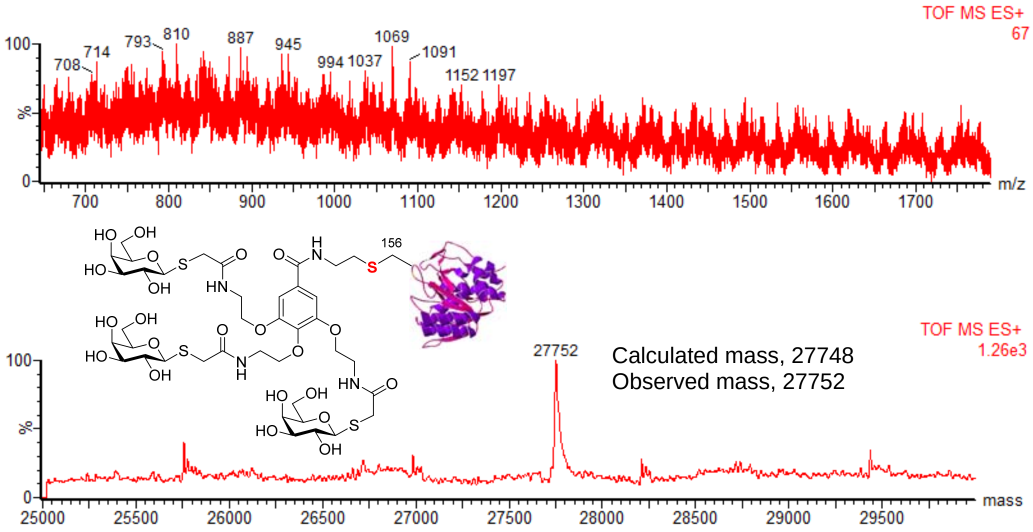

Figure S3. ESI–MS of the incubation of Gal$_3$-G-*S*-156SBL (**18**) with human plasma

Qβ virus-like particles (Qβ)[8]

Sequence of Qβ-M16Hag mutant (modified residue highlighted)

AKLETVTLGNIGKDGHagQTLVLNPRGVNPTNGVASLSQAGAVPALEKRVTVSVSQPSRN
RKNYKVQVKIQNPTACTANGSCDPSVTRQAYADVTFSFTQYSTDEERAFVRTELAALLAS
PLLIDAIDQLNPAY

Calculated average isotopic mass = 14105.93 Da

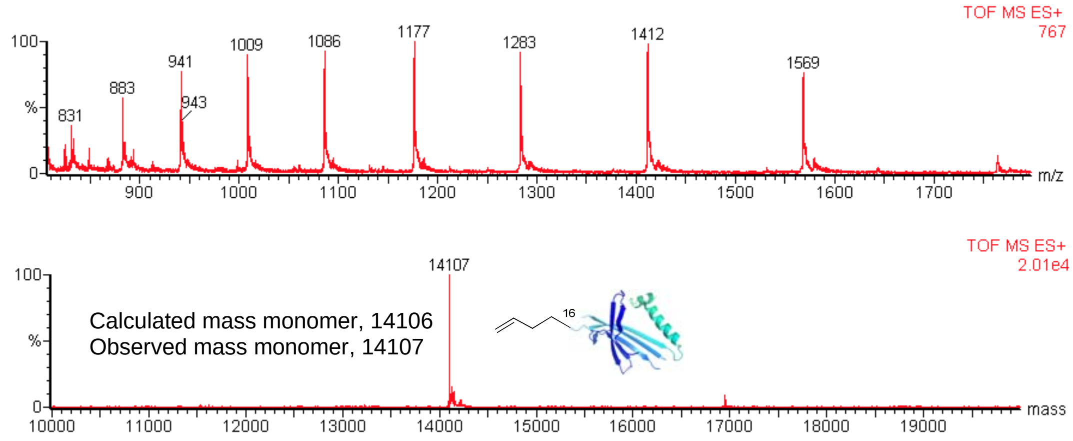

Figure S4. ESI–MS spectrum of Qβ-Hag16 (**14**) monomer

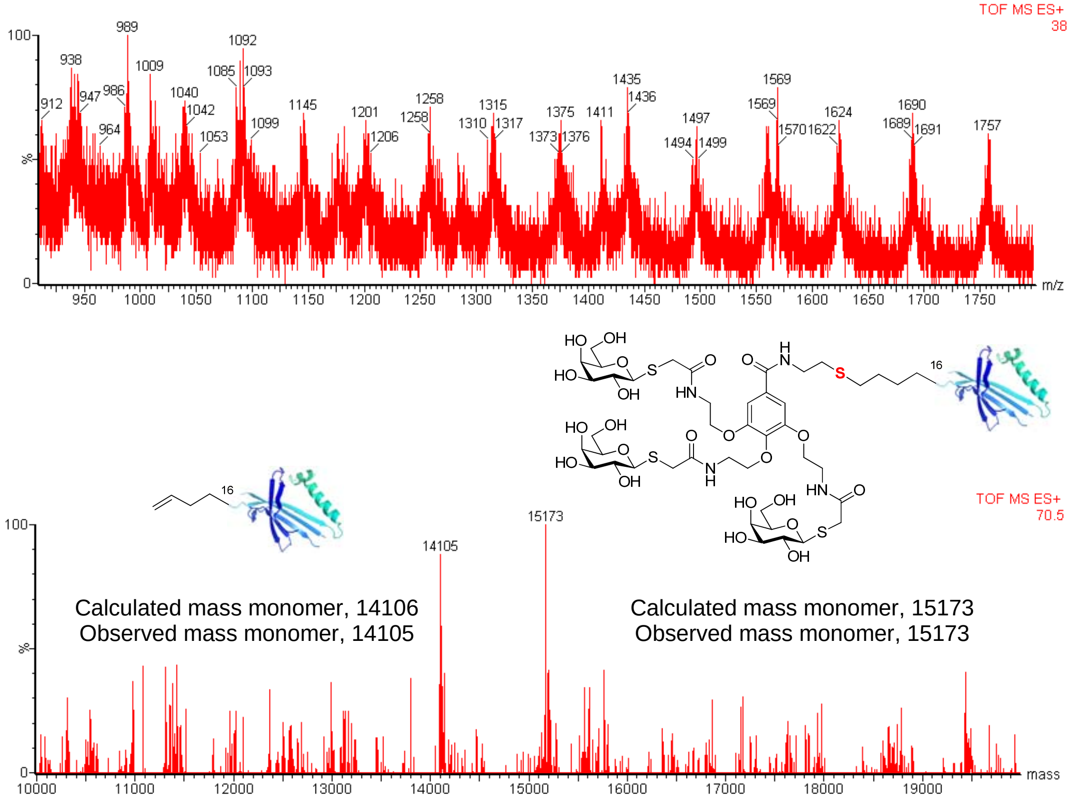

Figure S5. ESI–MS spectrum of the reaction of Qβ-Hag16 (**14**) with **1** in NH_4OAc buffer (250 mM, **pH 4.0**) at room temperature for 28 h (*ca.* 53% conv.)

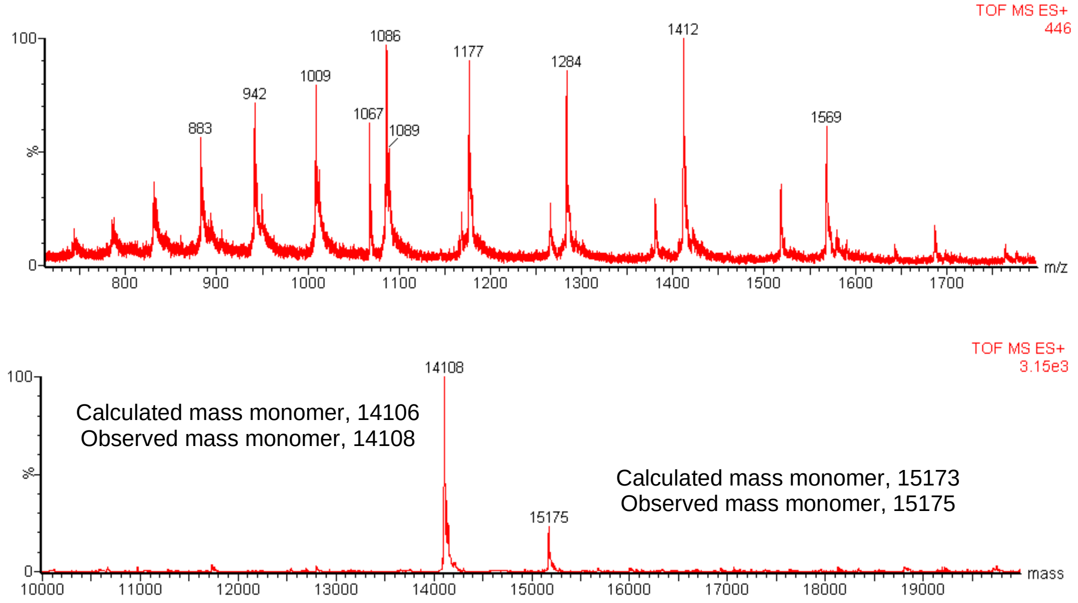

Figure S6. ESI–MS spectrum of the reaction of Qβ-Hag16 (**14**) with **1** in NH_4OAc buffer (250 mM, **pH 6.0**) at room temperature for 2 h (*ca.* 20% conv.)

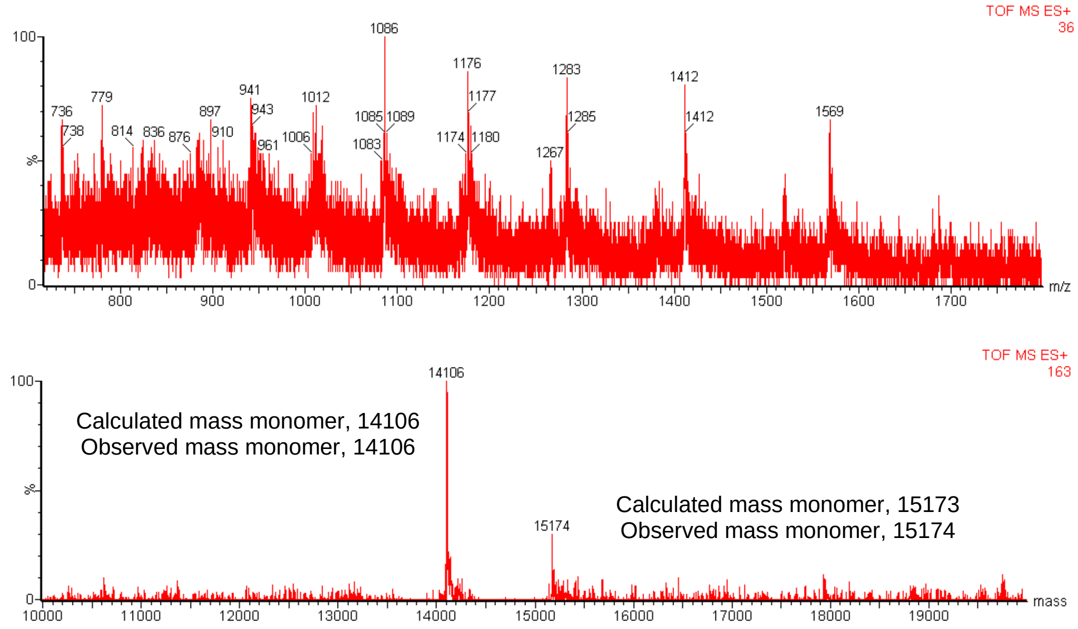

Figure S7. ESI–MS spectrum of the reaction of Qβ-Hag16 (**14**) with **1** in NH_4OAc buffer (250 mM, **pH 6.0**) at room temperature for 8 h (*ca.* 25% conv.)

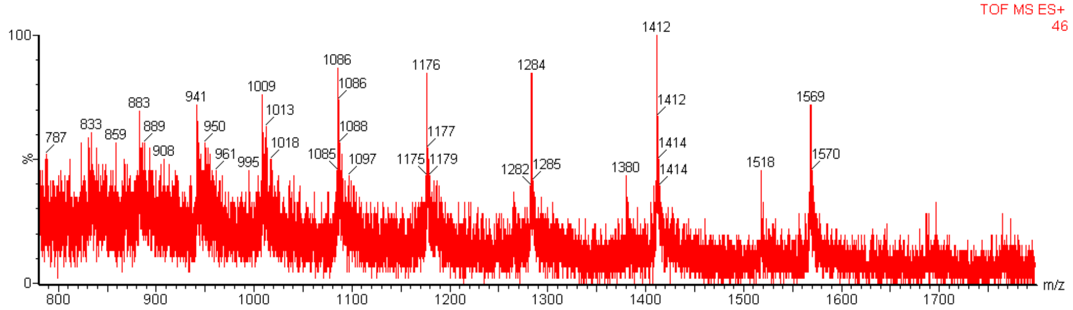

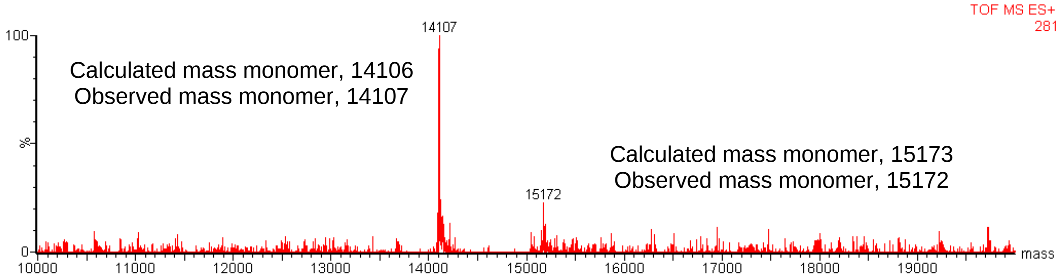

Figure S8. ESI–MS spectrum of the reaction of Qβ-Hag16 (**14**) with **1** in NH_4OAc buffer (250 mM, **pH 6.0**) at room temperature for 30 h (*ca.* 20% conv.)

β-Glycosidase from *Sulfolobus solfataricus* (*Ss*βG)[12]

Sequence of *Ss*βG M21I M43Aha M73I M148I M204I M236I M275I M280I C344S M383I M439I mutant (modified residue highlighted)

GHHHHHHHSFPNSFRFGWSQAGFQSEIGTPGSEDPNTDWYKWVHDPENAhaAAGLVSGD
LPENGPGYWGNYKTFHDNAQKIGLKIARLNVEWSRIFPNPLPRPQNFDESKQDVTEVEINE
NELKRLDEYANKDALNHYREIFKDLKSRGLYFILNIYHWPLPLWLHDPIRVRRGDFTGPSG
WLSTRTVYEFARFSAYIAWKFDDLVDEYSTINEPNVVGGLGYVGVKSGFPPGYLSFELSR
RAIYNIIQAHARAYDGIKSVSKKPVGIIYANSSFQPLTDKDIEAVEIAENDNRWWFFDAIIR
GEITRGNEKIVRDDLKGRLDWIGVNYYTRTVVKRTEKGYVSLGGYGHGSERNSVSLAGL
PTSDFGWEFFPEGLYDVLTKYWNRYHLYIYVTENGIADDADYQRPYYLVSHVYQVHRAI
NSGADVRGYLHWSLADNYEWASGFSIRFGLLKVDYNTKRLYWRPSALVYREIATNGAIT
DEIEHLNSVPPVKPLRH

Calculated average isotopic mass = 57230.87 Da

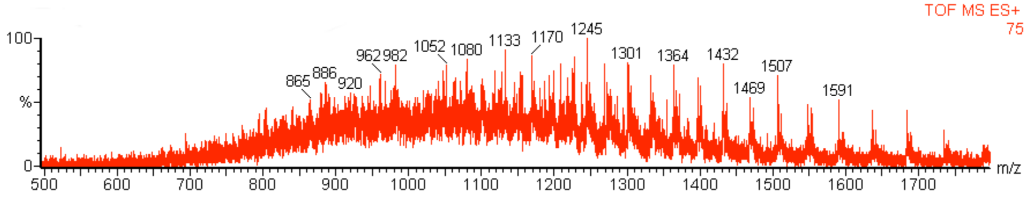

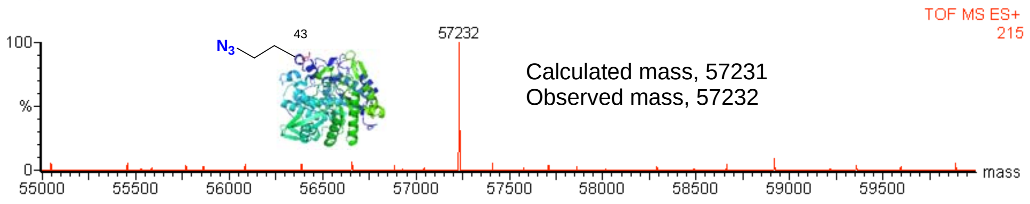

Figure S9. ESI–MS spectrum of *Ss*βG-Aha43 (**15**)

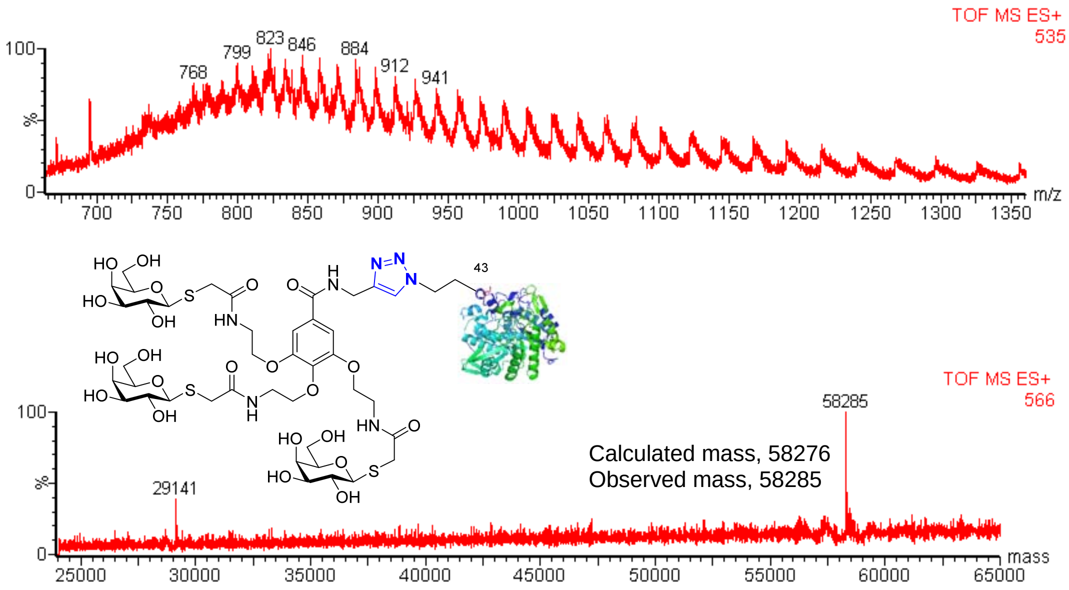

Figure S10. ESI–MS spectrum of Gal3-G-*triazole*-43*Ss*βG (**20**)

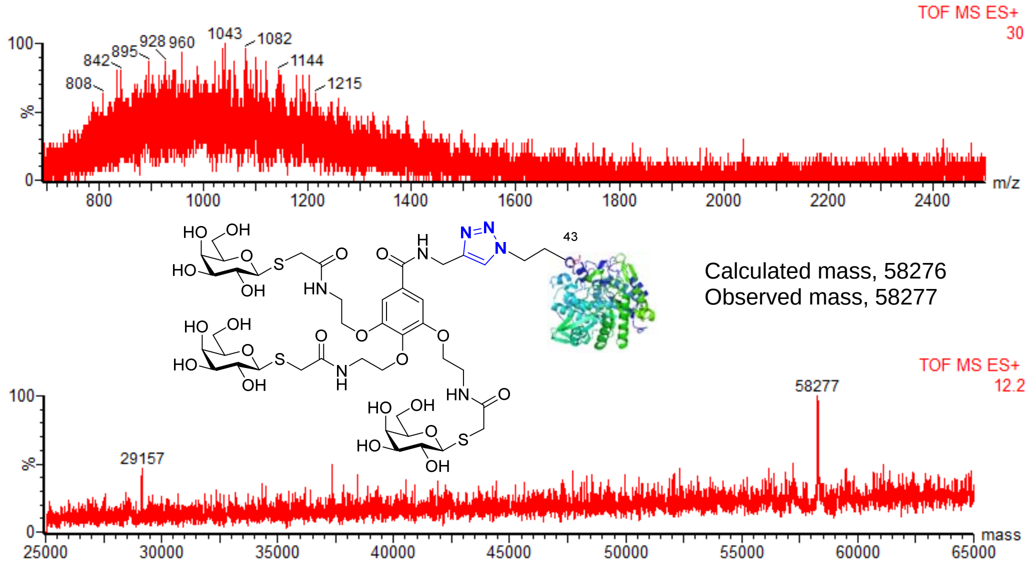

Figure S11. ESI–MS of the incubation of Gal3-G-*triazole*-43*Ss*βG (**20**) with human plasma

Np276 from *Nostoc punctiforme* (Np276)[10,12]

Sequence of Np276 M21I M61Aha I101C K25A K94A K123A T201K mutant (modified residue highlighted)

GSSHHHHHHSSGLVPRGSHIDVEALRQLYAAGERDFSIVDLRGAVLENINLSGAILHGAAhaLD
EANLQQANLSRADLSGATLNGADLRGANLSAADLSDACLDNAILEGAILDEAVLNQANLAAA
NLEQAILSHANIREADLSEANLEAADLSGADLAIADLHQANLHQAALERANLTGANLEDANL
EGTILEGGNNNLAK

Calculated average isotopic mass = 20750.72 Da

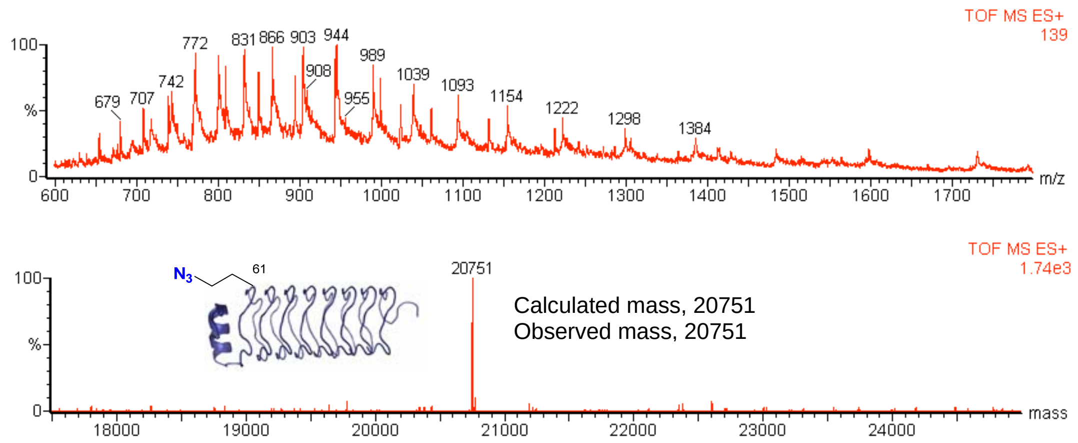

Figure S12. ESI–MS spectrum of Np276-Aha61 (**16**)

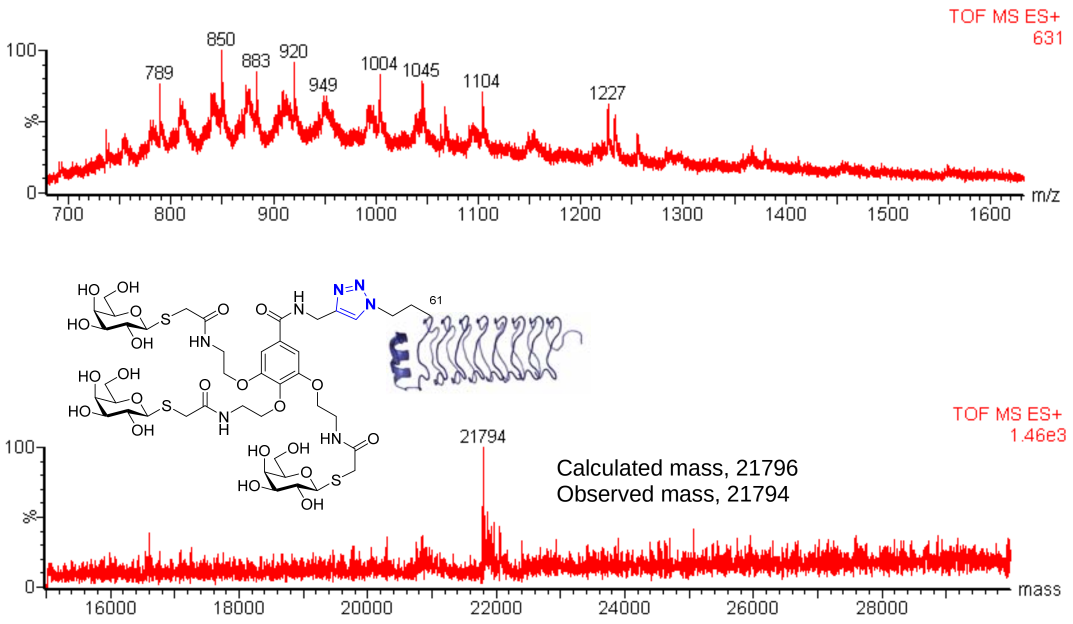

Figure S13. ESI–MS spectrum of Gal3-G-*triazole*-61Np276 (**21**)

β-Glycosidase from *Sulfolobus solfataricus* (*Ss*βG)[13]

Sequence of *Ss*βG (M1Hpg) M21I M43Hpg M73I M148I M204I M236I M275I M280I C344S M383I M439I mutant (modified residue highlighted)

(Hpg)GHHHHHHHSFPNSFRFGWSQAGFQSEIGTPGSEDPNTDWYKWVHDPENHpgAAGL
VSGDLPENGPGYWGNYKTFHDNAQKIGLKIARLNVEWSRIFPNPLPRPQNFDESKQDVTE
VEINENELKRLDEYANKDALNHYREIFKDLKSRGLYFILNIYHWPLPLWLHDPIRVRRGDF
TGPSGWLSTRTVYEFARFSAYIAWKFDDLVDEYSTINEPNVVGGLGYVGVKSGFPPGYLS
FELSRRAIYNIIQAHARAYDGIKSVSKKPVGIIYANSSFQPLTDKDIEAVEIAENDNRWWFF
DAIIRGEITRGNEKIVRDDLKGRLDWIGVNYYTRTVVKRTEKGYVSLGGYGHGSERNSVS
LAGLPTSDFGWEFFPEGLYDVLTKYWNRYHLYIYVTENGIADDADYQRPYYLVSHVYQV
HRAINSGADVRGYLHWSLADNYEWASGFSIRFGLLKVDYNTKRLYWRPSALVYREIATN
GAITDEIEHLNSVPPVKPLRH

Calculated average isotopic mass = 57323.03 Da (2xHpg)
57213.87 Da (1xHpg)

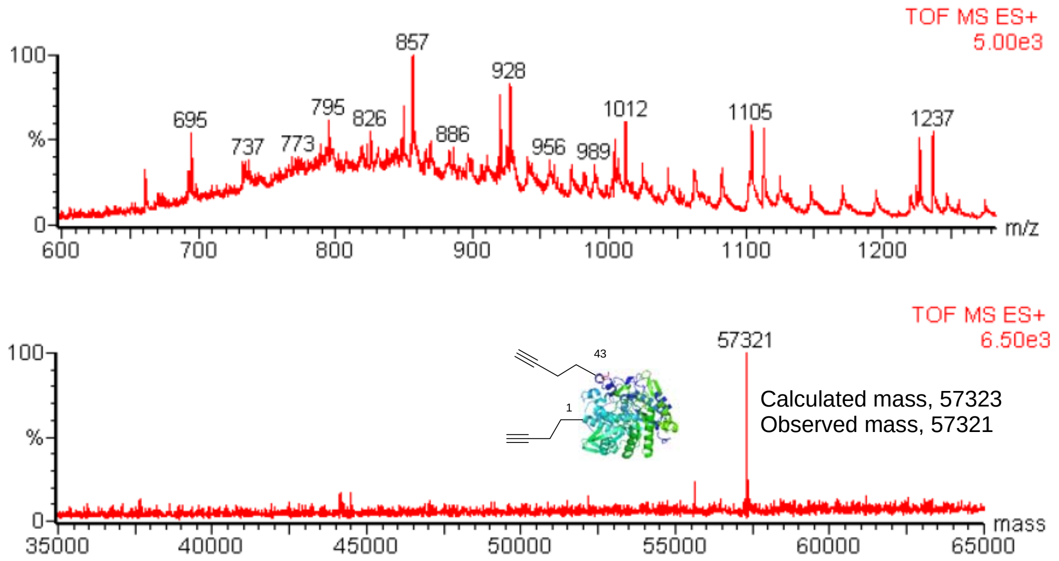

Figure S14. ESI–MS spectrum of *Ss*βG-Hpg1-Hpg43 (**17a**)

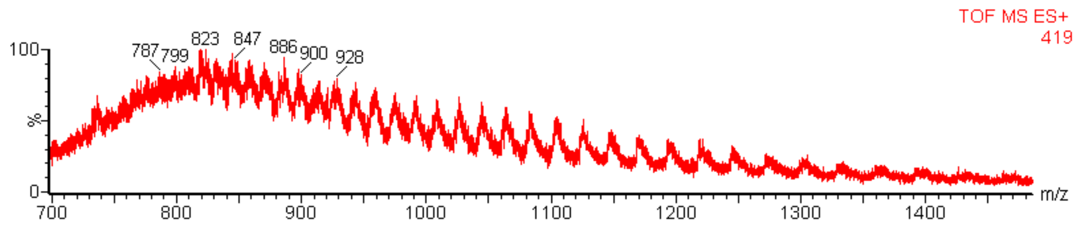

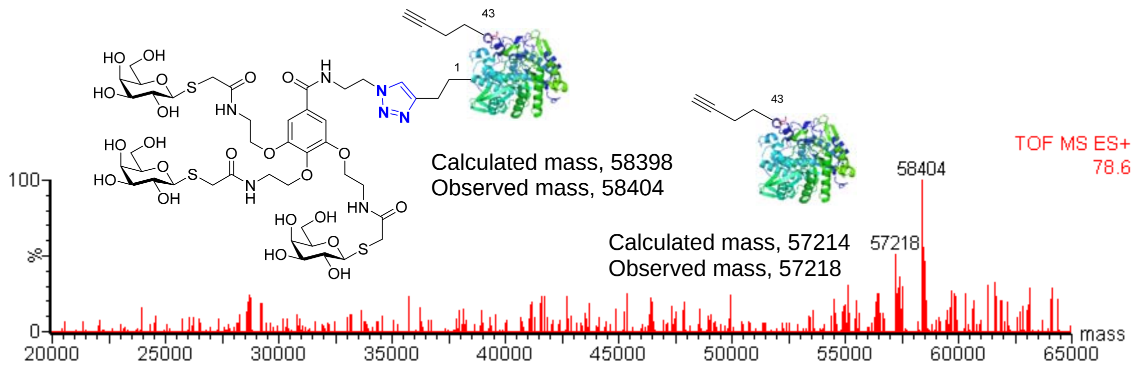

Figure S15. ESI–MS spectrum of Gal3-G-*triazole*-1*Ss*βG (**22**) showing residual, post-translationally modified *Ss*βG-Hpg43 (**17b**) resulting from *N*-terminal Hpg excision in **17a**

3. SDS-PAGE Gels

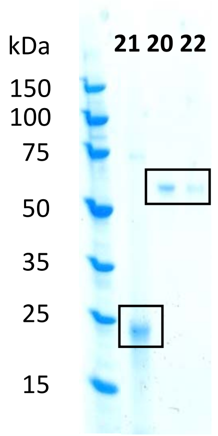

Figure S16. SDS-PAGE analysis of modified proteins Gal3-G-*triazole*-43*Ss*βG (**20**), Gal3-G-*triazole*-61Np276 (**21**), and Gal3-G-*triazole*-1*Ss*βG (**22**) in MES SDS running buffer

4. NMR Spectra

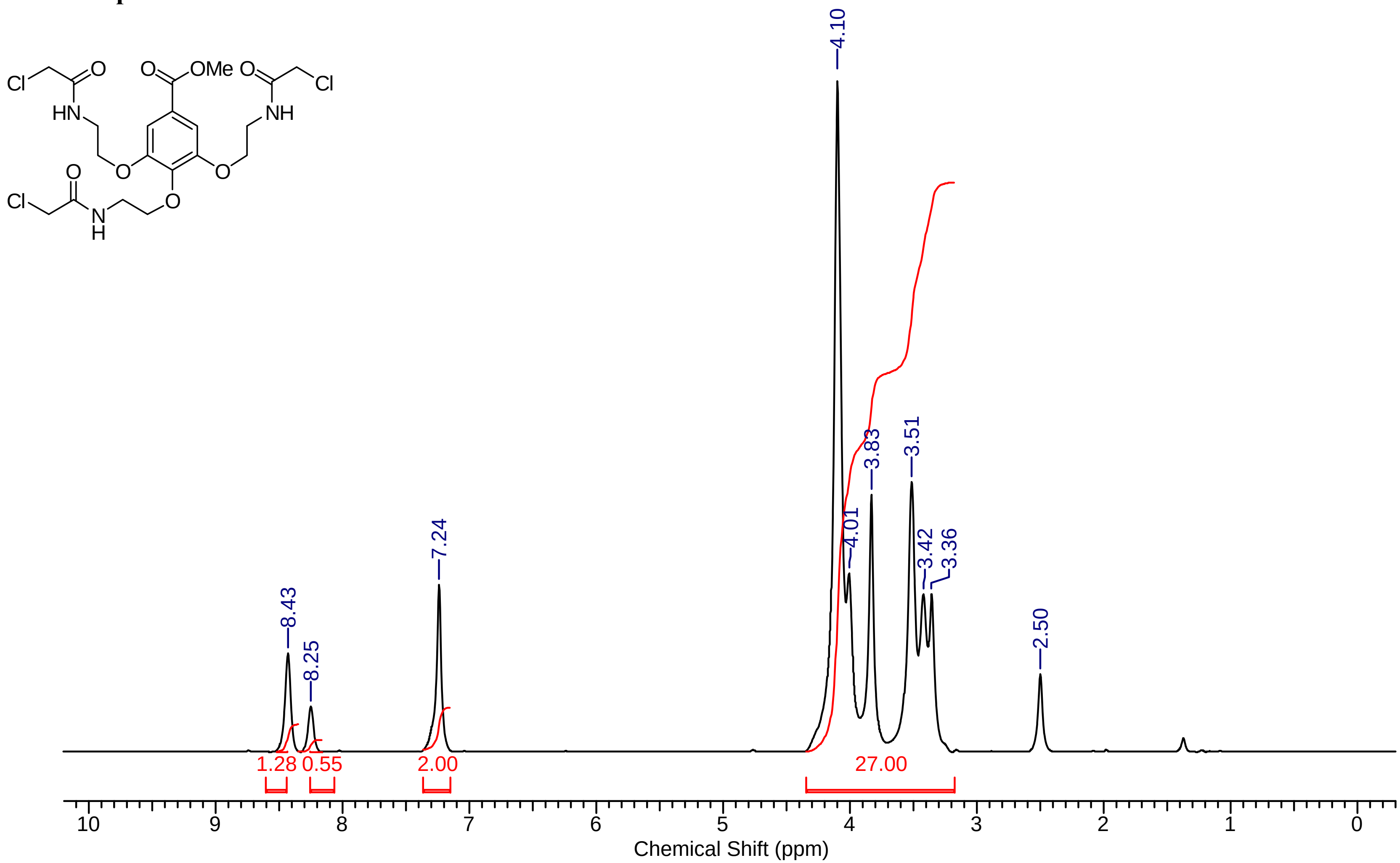

Figure S17. ^{1}H NMR (400 MHz, DMSO-d_6) of **7**

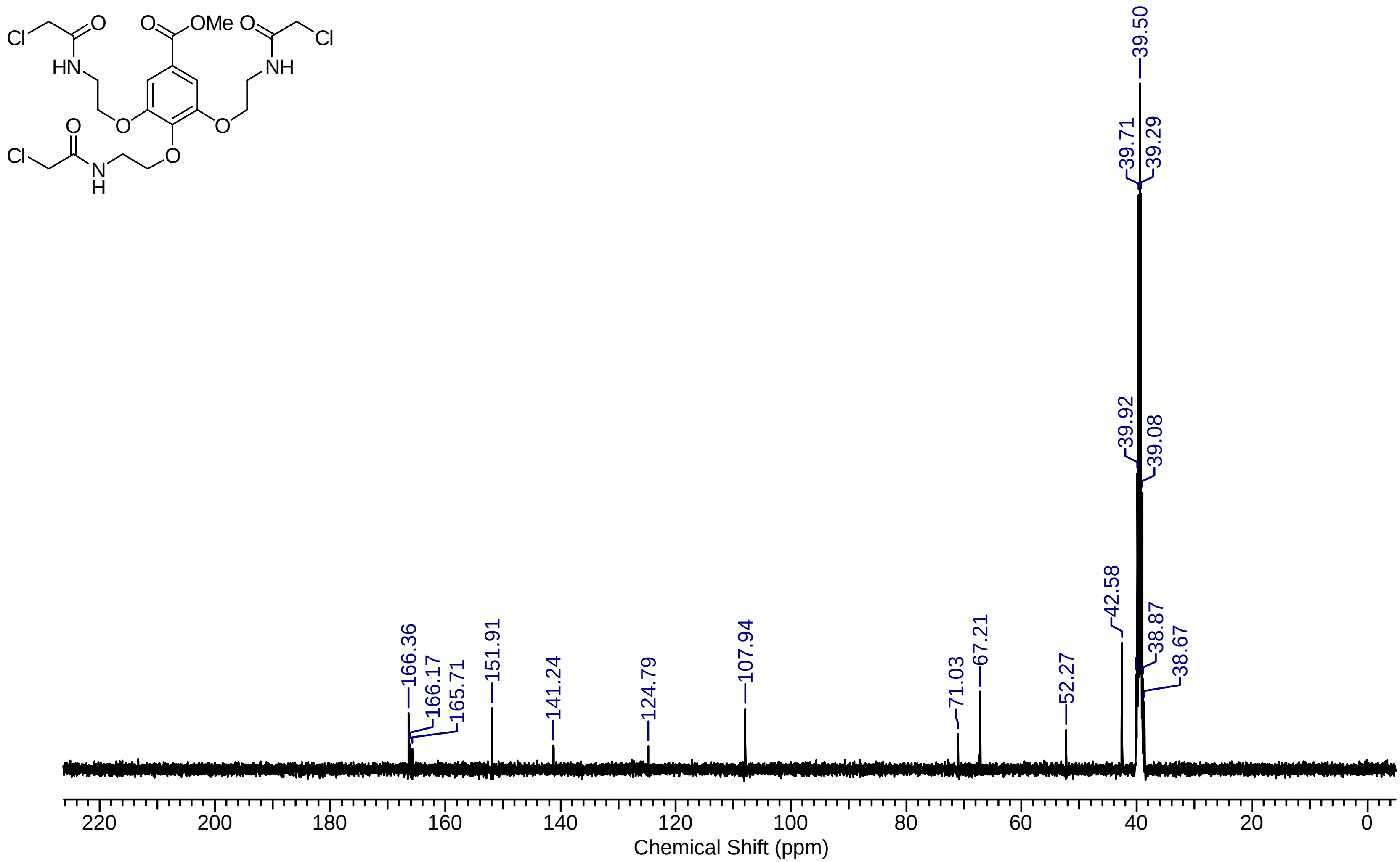

Figure S18. ^{13}C NMR (100.6 MHz, DMSO-d_6) of **7**

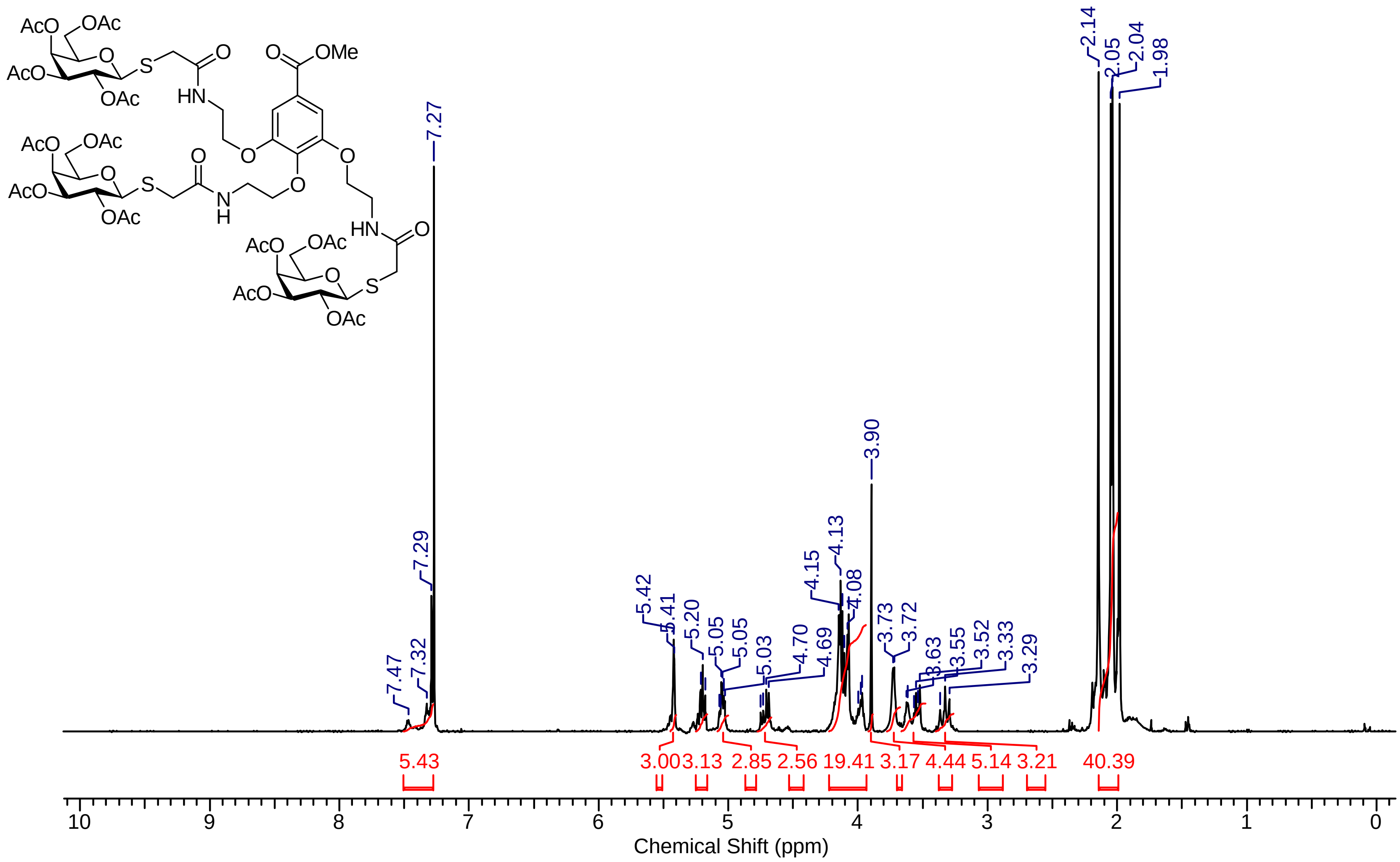

Figure S19. ^{1}H NMR (500 MHz, $CDCl_3$) of **10**

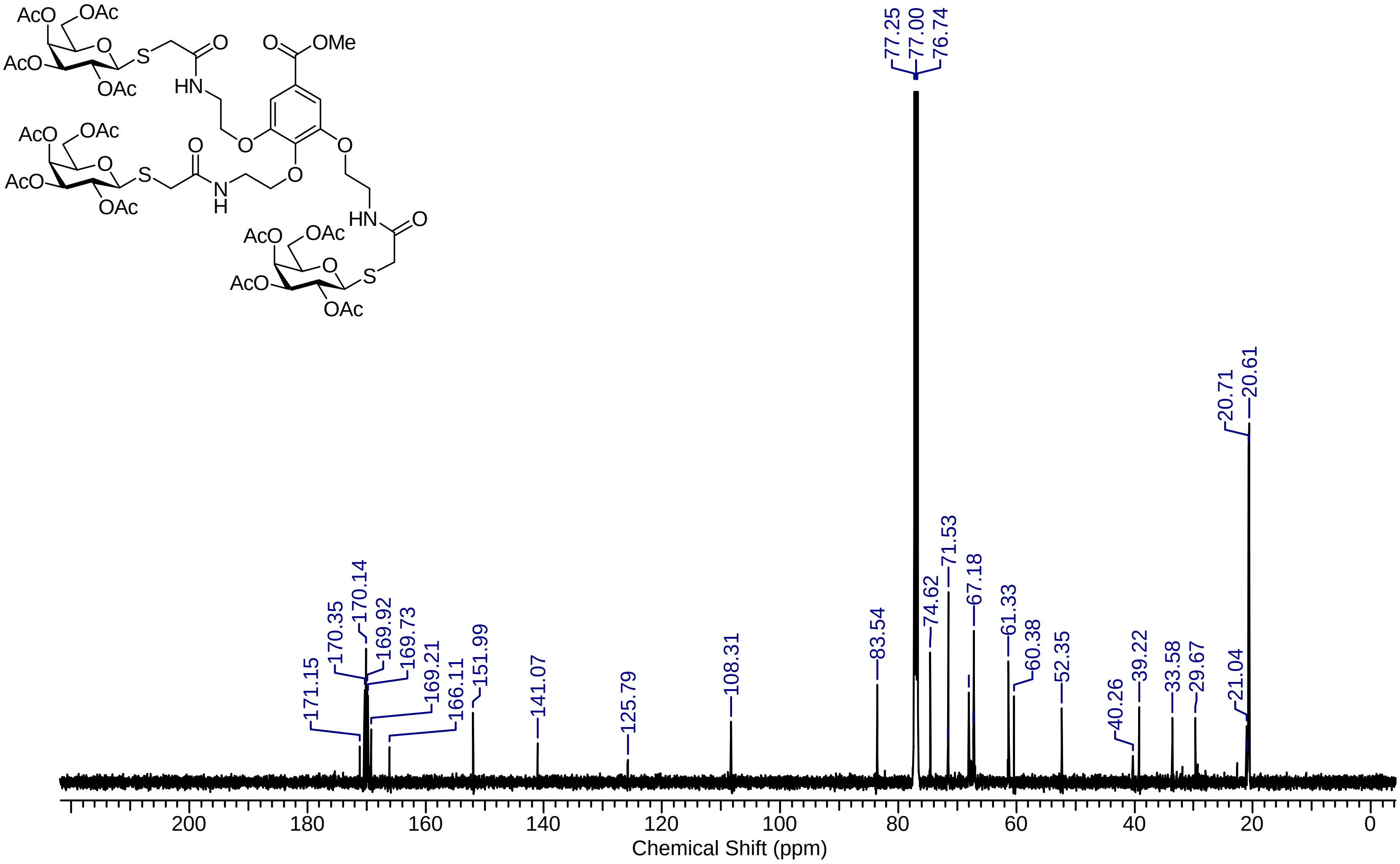

Figure S20. ^{13}C NMR (125.8 MHz, $CDCl_3$) of **10**

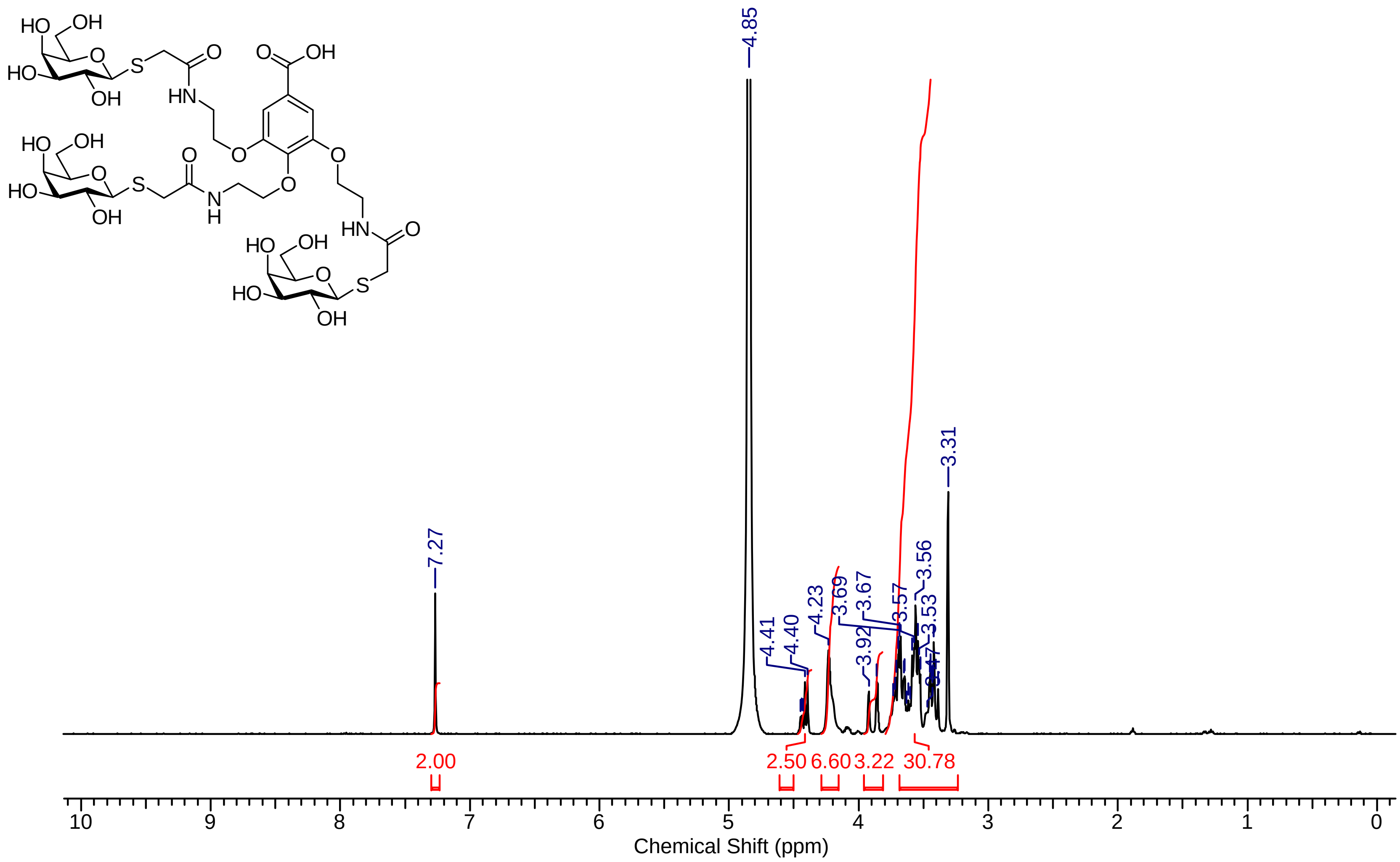

Figure S21. ^{1}H NMR (500 MHz, CD_3OD) of **11**

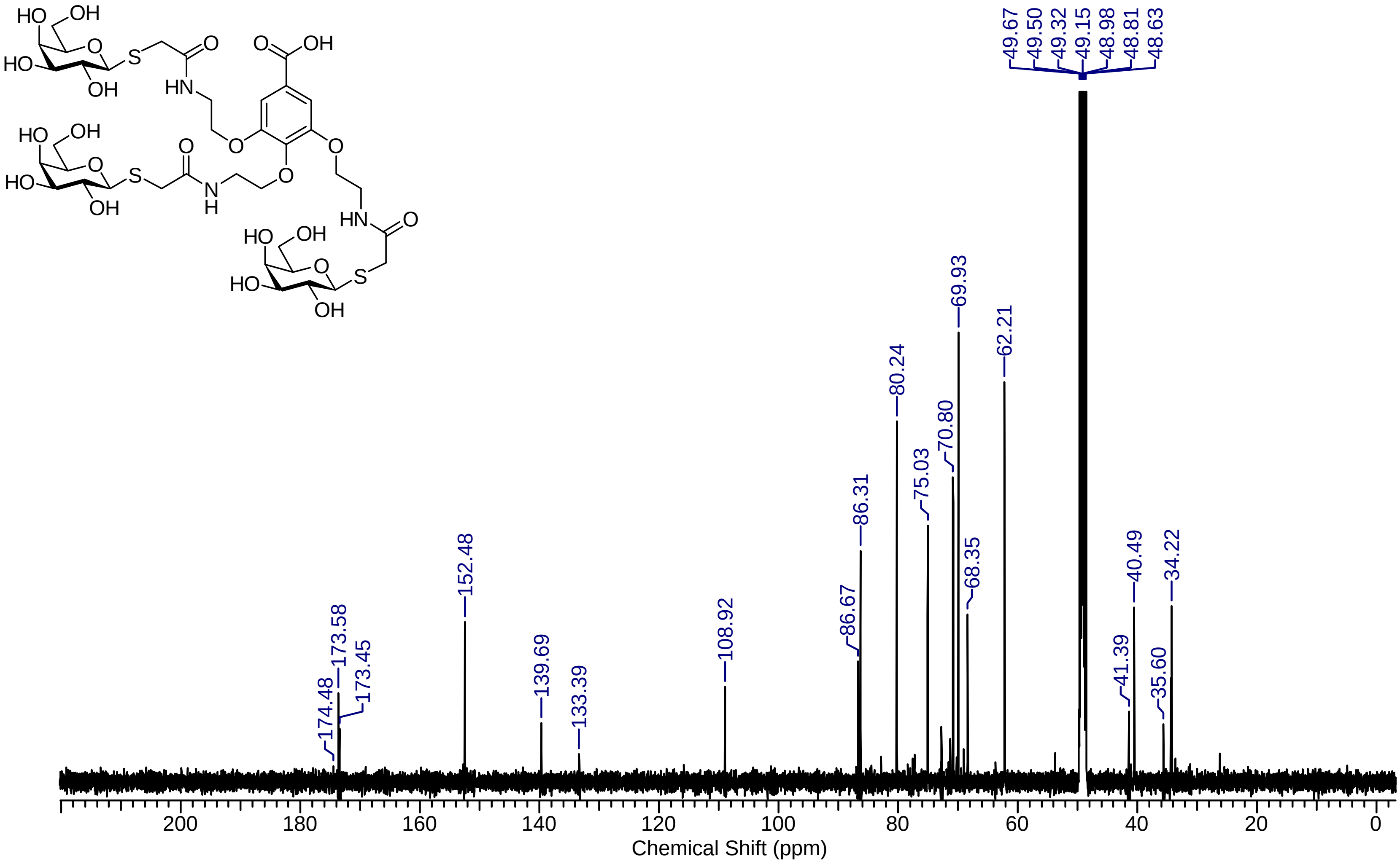

Figure S22. ^{13}C NMR (125.8 MHz, CD_3OD) of **11**

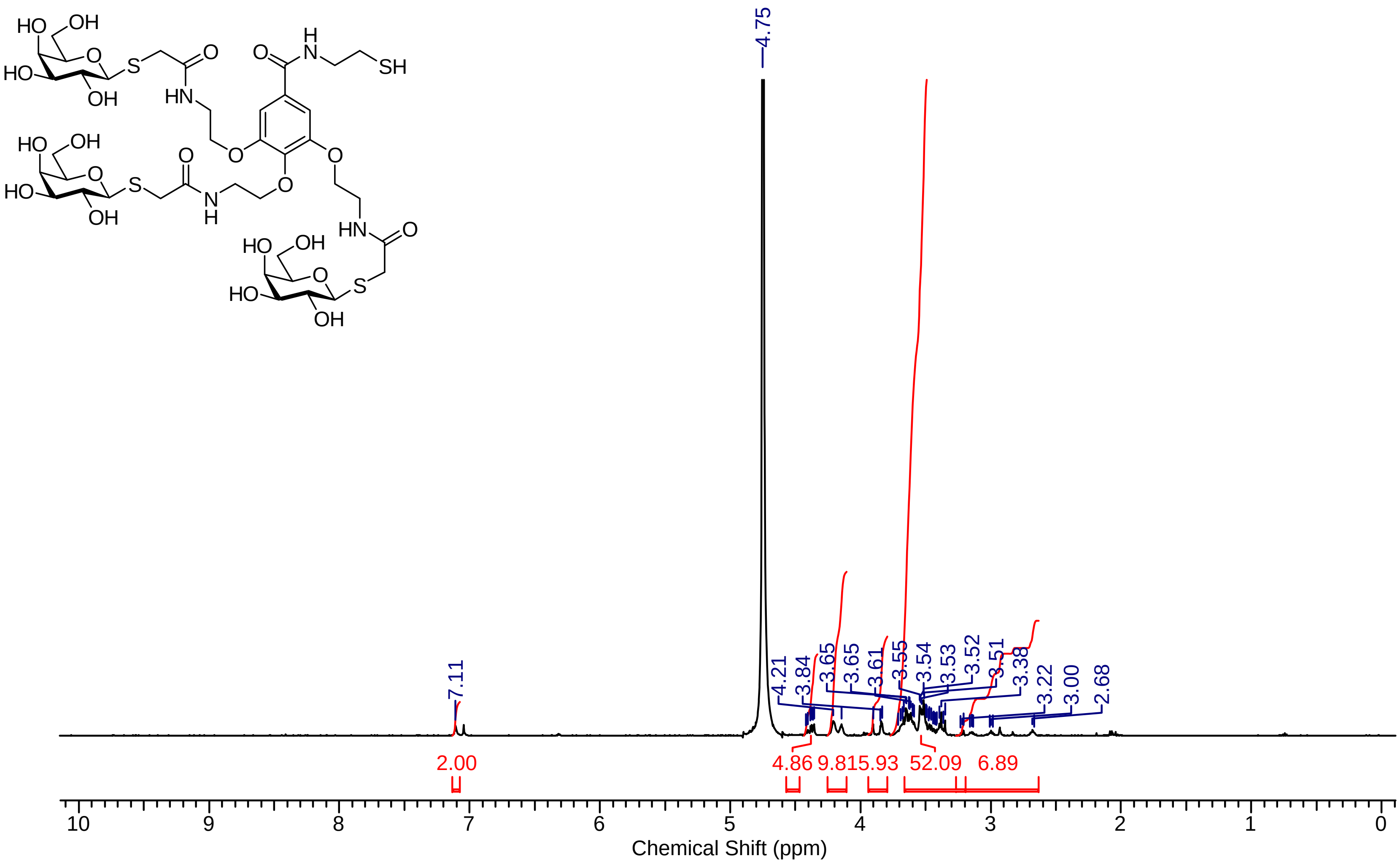

Figure S23. ^{1}H NMR (500 MHz, D_2O) of **1**

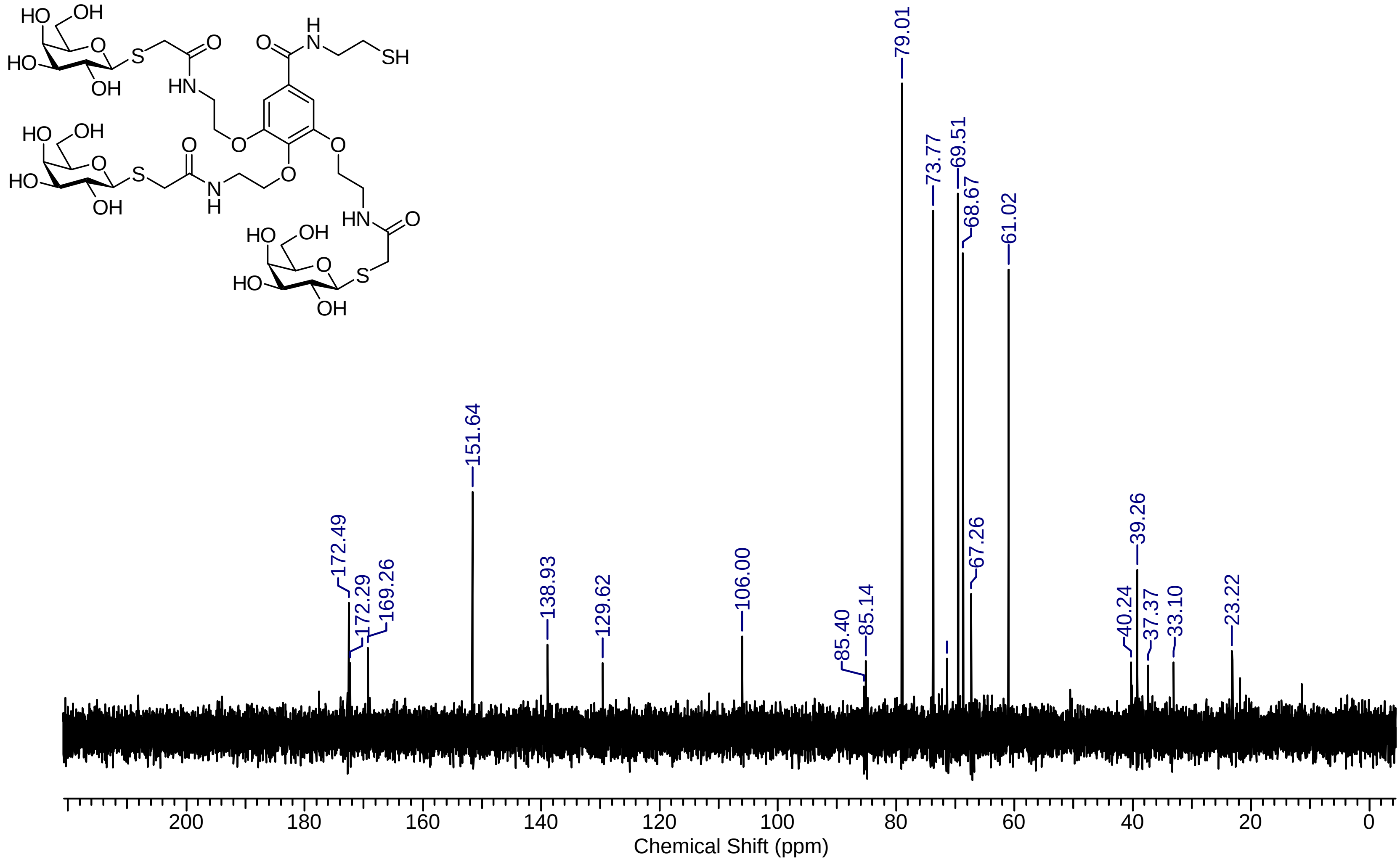

Figure S24. ^{13}C NMR (125.8 MHz, D_2O) of **1**

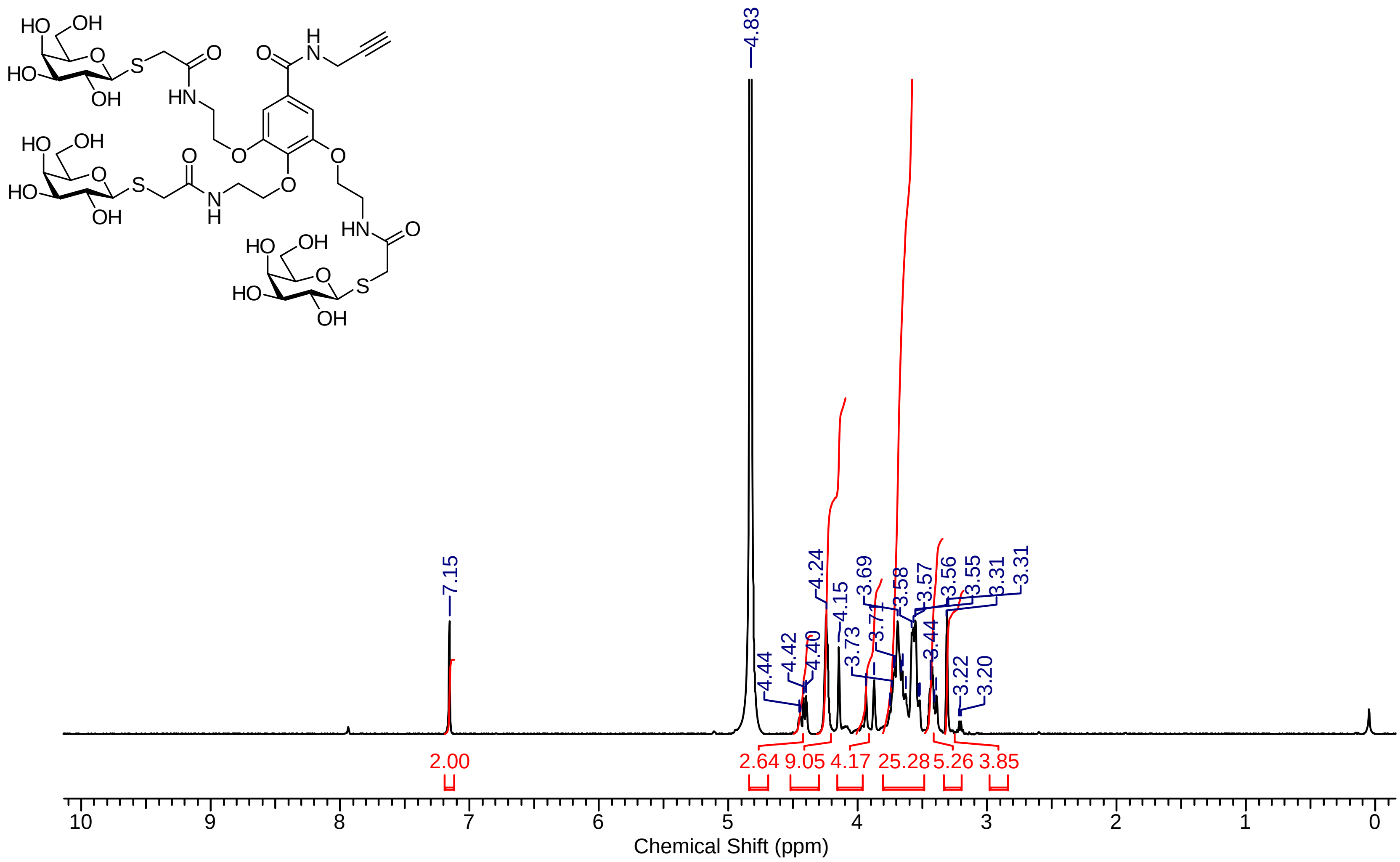

Figure S25. ¹H NMR (500 MHz, CD_3OD) of **2**

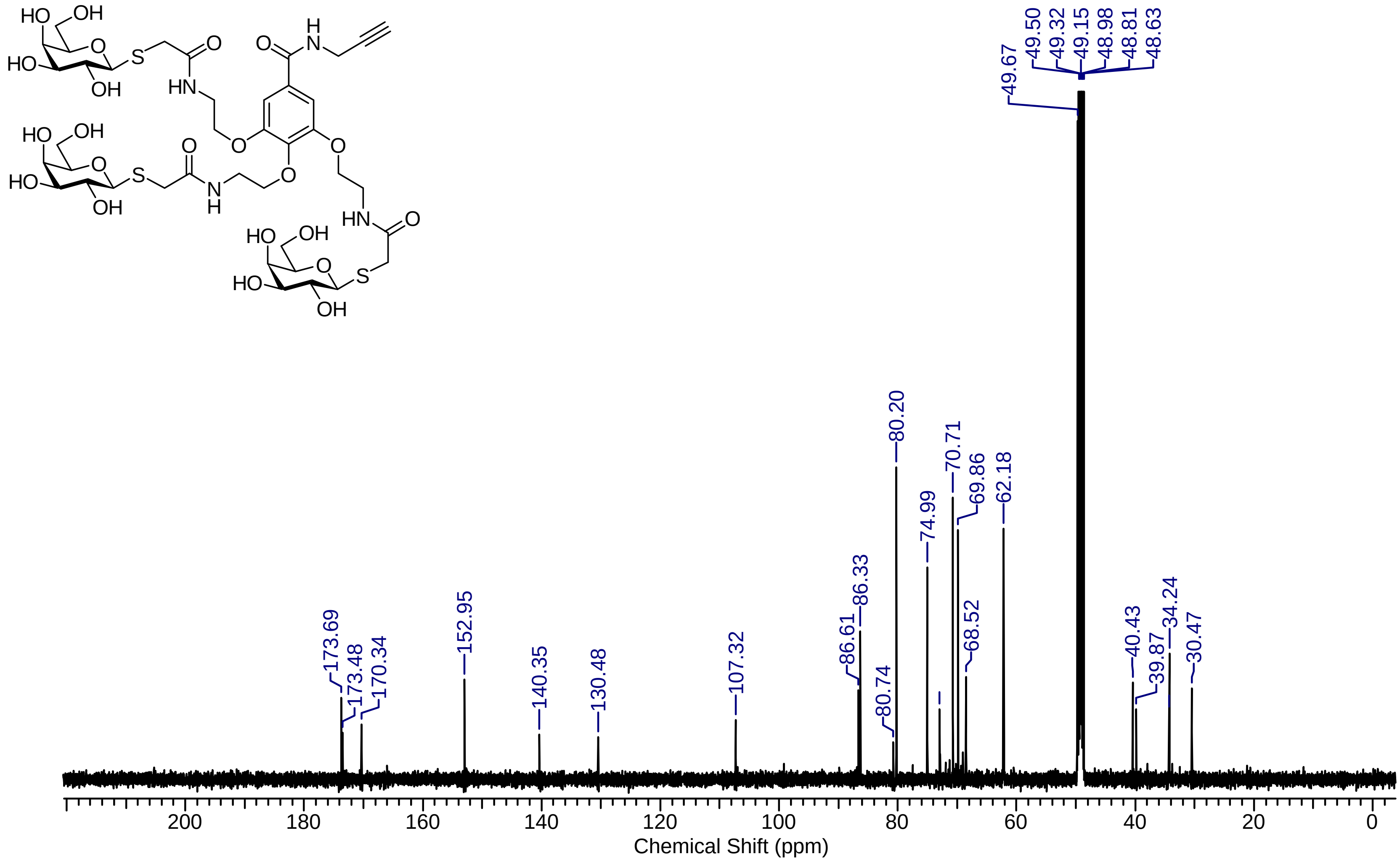

Figure S26. ^{13}C NMR (125.8 MHz, CD_3OD) of **2**

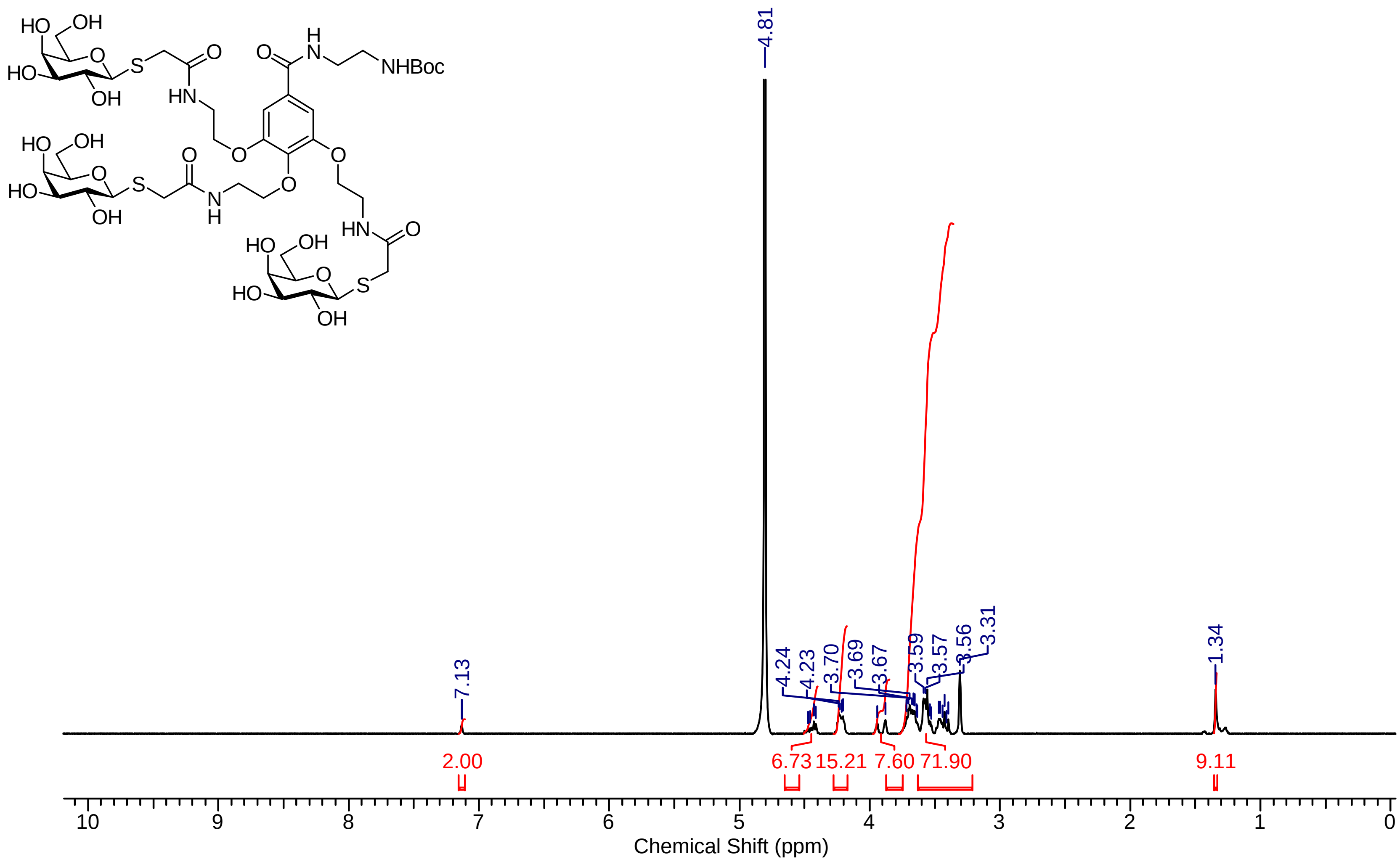

Figure S27. ^{1}H NMR (500 MHz, CD_3OD) of **12**

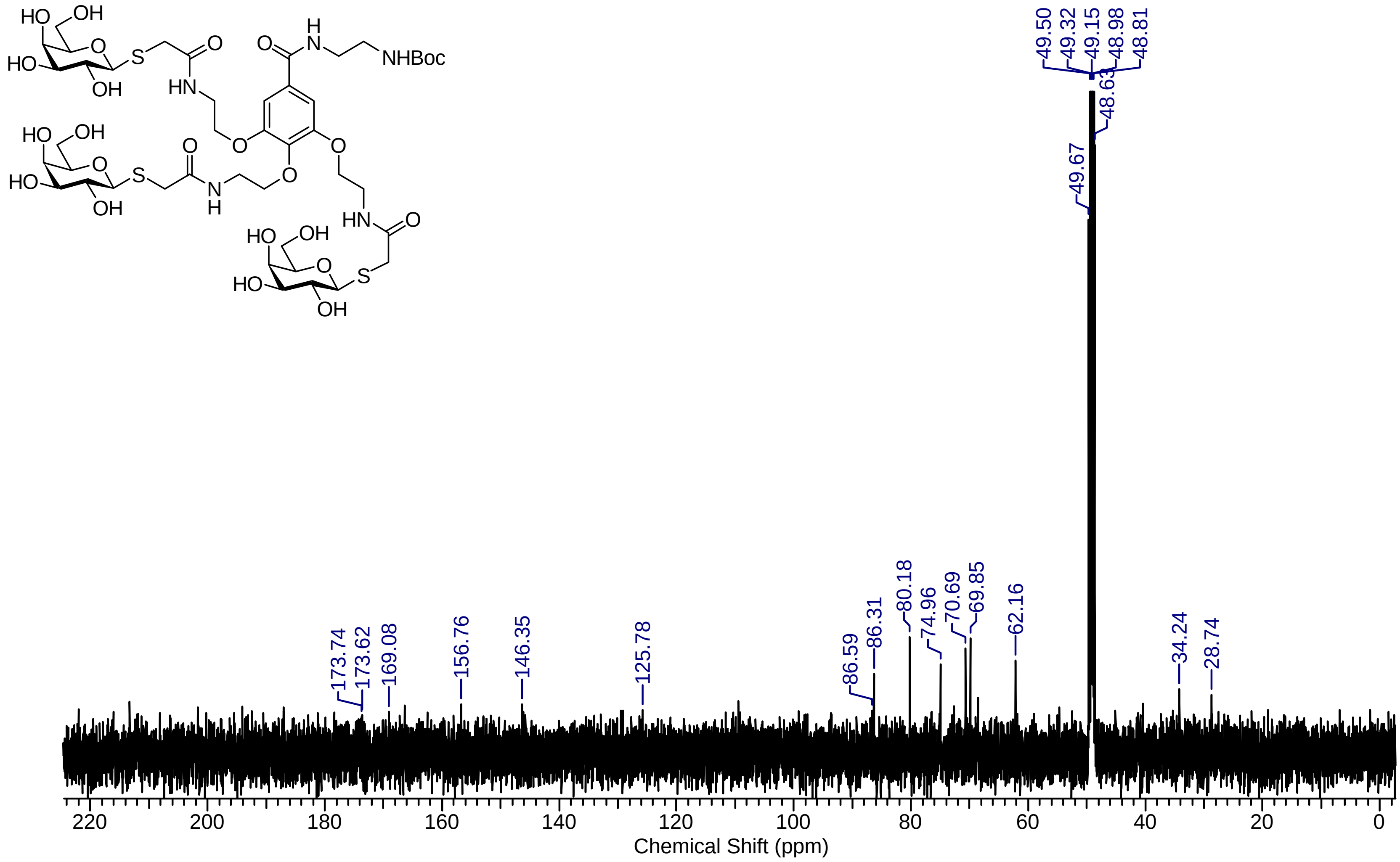

Figure S28. ^{13}C NMR (125.8 MHz, CD_3OD) of **12**

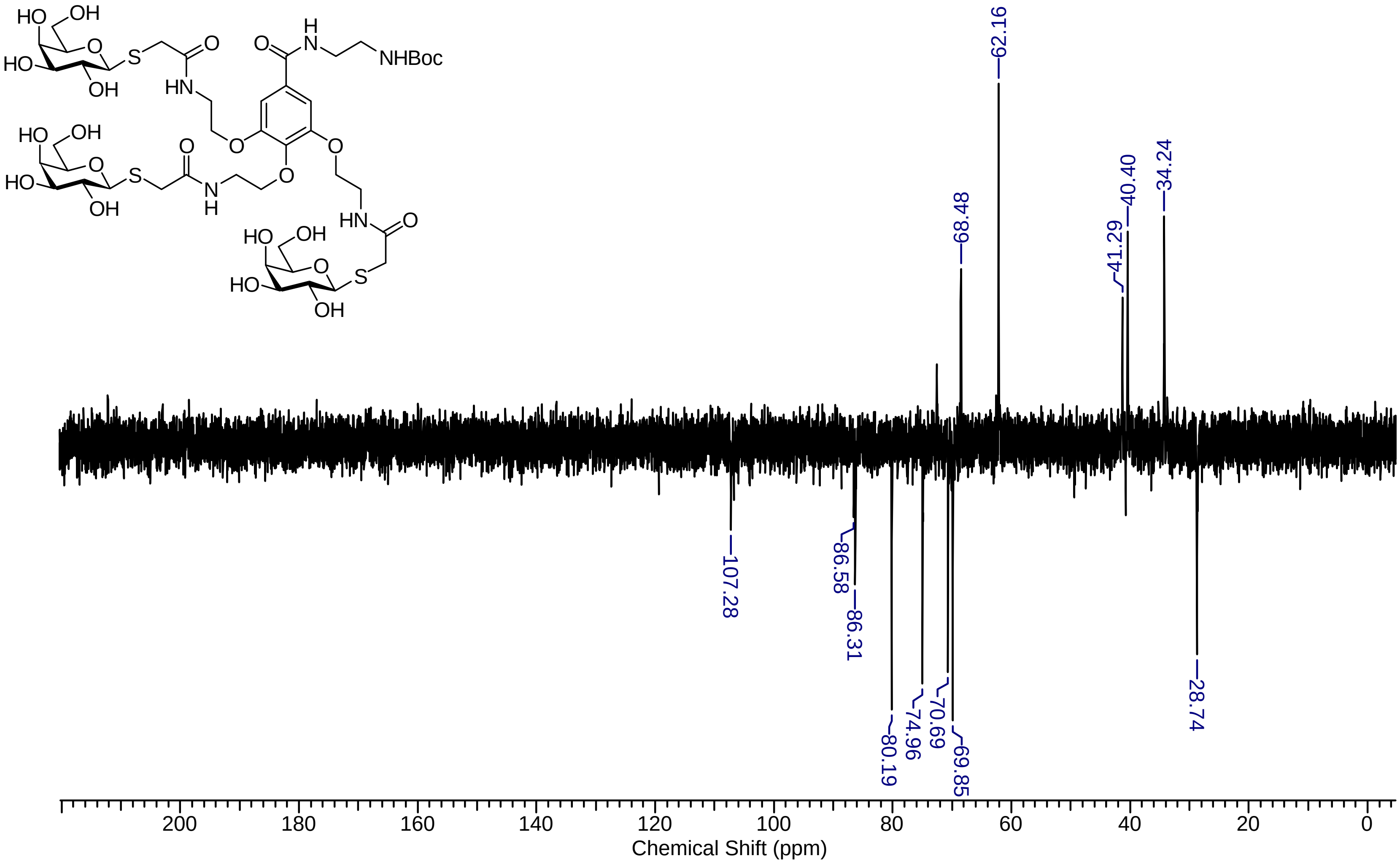

Figure S29. DEPT 135 (500 MHz, CD_3OD) of **12**

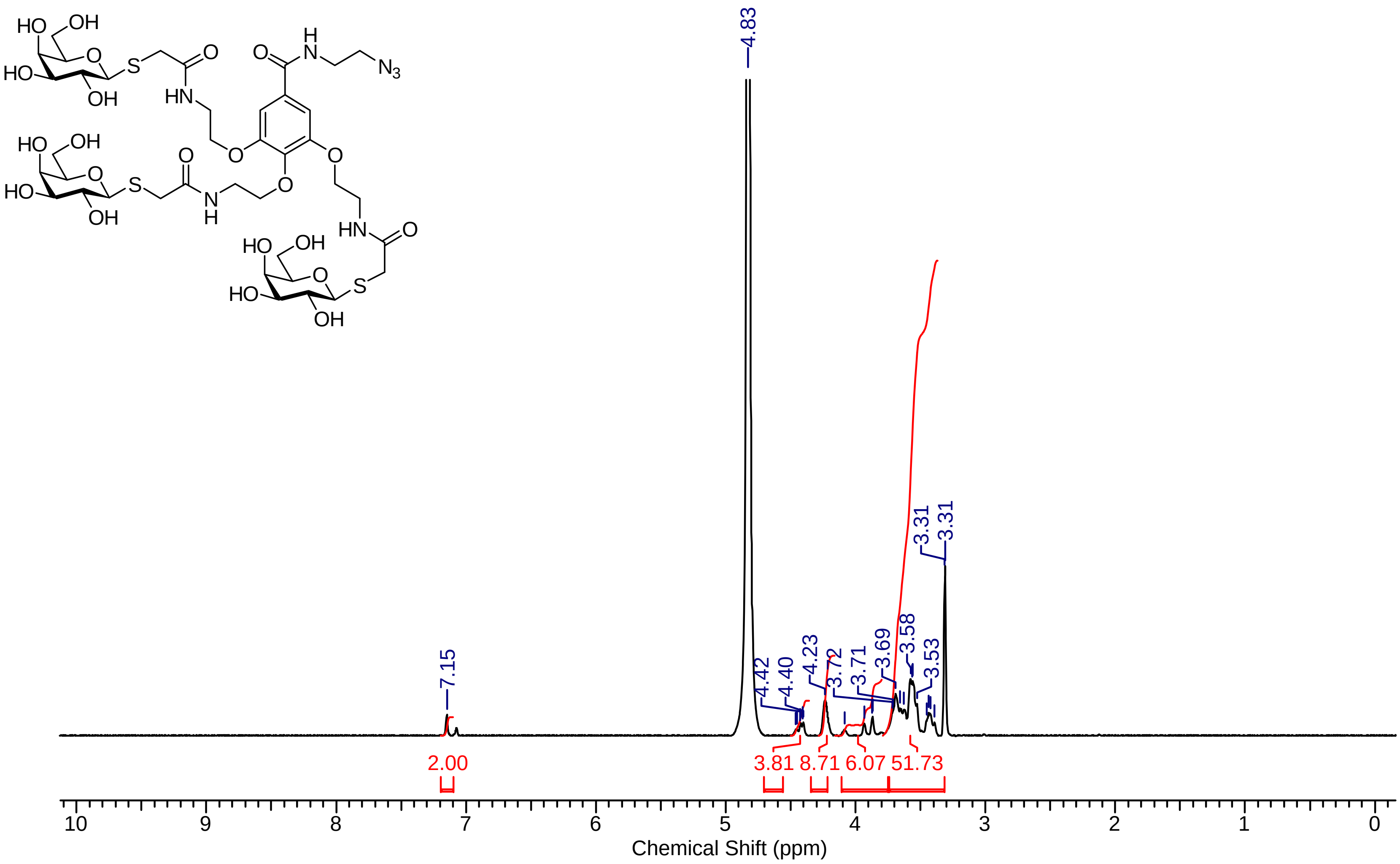

Figure S30. ¹H NMR (500 MHz, CD_3OD) of **3**

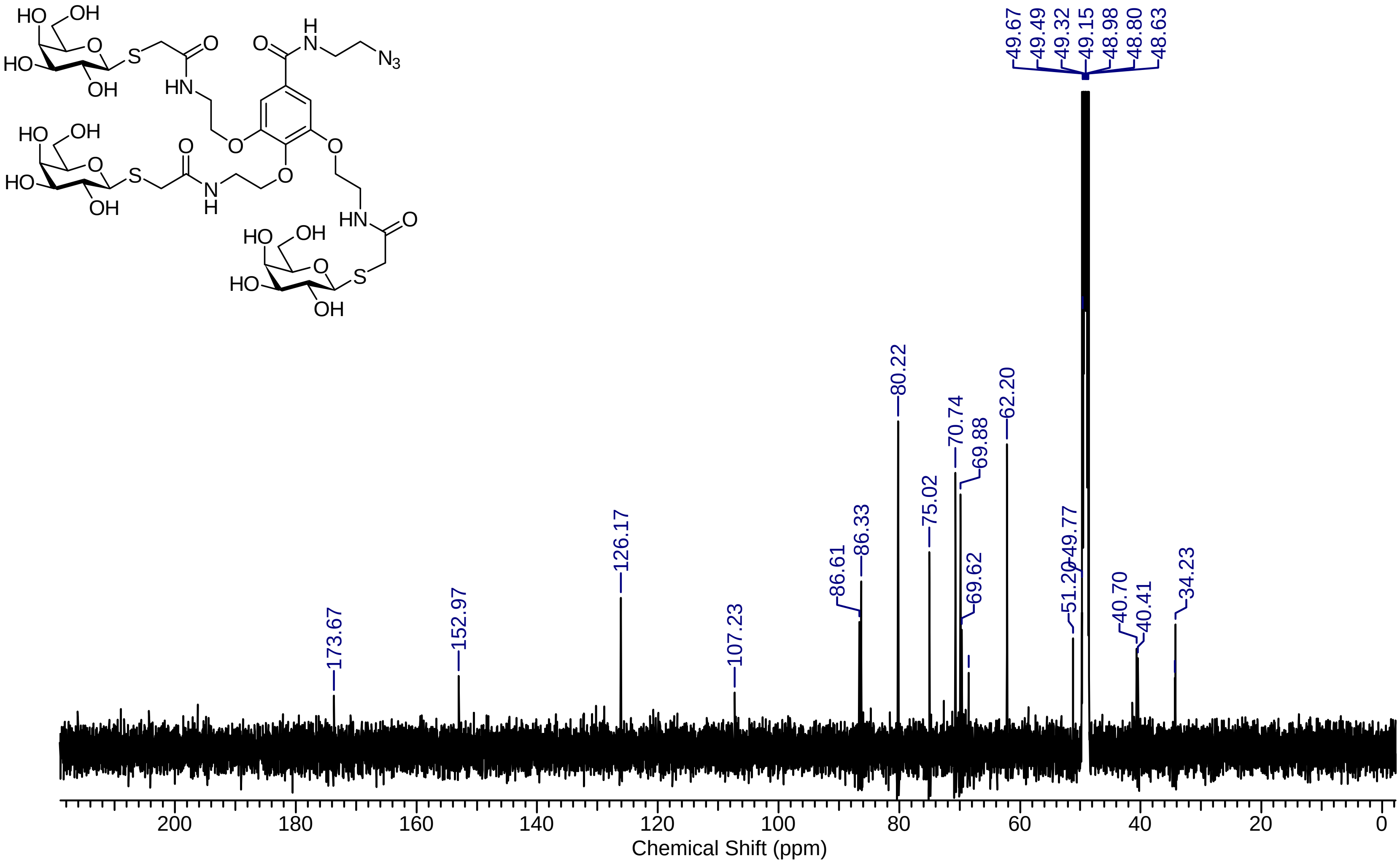

Figure S31. ^{13}C NMR (125.8 MHz, CD_3OD) of **3**

5. References

(1) Roy, R.; Park, W. K. C.; Wu, Q.; Wang, S-.N. Synthesis of hyper-branched dendritic lactosides. *Tetrahedron Lett.* **1995**, *36*, 4377–4380.

(2) Gassman, P. G.; Schenk, W. N. A general procedure for the base-promoted hydrolysis of hindered esters at ambient temperatures. *J. Org. Chem.* **1977**, *42*, 918–920.

(3) Stanton, M. G.; Gagné, M. R. The remarkable catalytic activity of alkali-metal alkoxide clusters in the ester interchange reaction. *J. Am. Chem. Soc.* **1997**, *119*, 5075–5076.

(4) Bartlett, P. A.; Johnson, W. S. An improved reagent for the o-alkyl cleavage of methyl esters by nucleophilic displacement. *Tetrahedron Lett.* **1970**, *11*, 4459–4462.

(5) Lin, Y. A.; Boutureira, O.; Lercher, L.; Bhushan, B.; Paton, R. S.; Davis, B. G. Rapid cross-metathesis for reversible protein modifications via chemical access to *Se*-allyl-selenocysteine in proteins. *J. Am. Chem. Soc.* **2013**, *135*, 12156–12159.

(6) Boutureira, O.; Bernardes, G. J. L.; Fernández-González, M.; Anthony, D. C.; Davis, B. G. Selenenylsulfide-linked homogeneous glycopeptides and glycoproteins: synthesis of human "hepatic Se metabolite A." *Angew. Chem. Int. Ed.* **2011**, *51*, 1432–1436.

(7) Chalker, J. M.; Gunnoo, S. B.; Boutureira, O.; Gerstberger, S. C.; Fernández-González, M.; Bernardes, G. J. L.; Griffin, L.; Hailu, H.; Schofield, C. J.; Davis, B. G. Methods for converting cysteine to dehydroalanine on peptides and proteins. *Chem. Sci.* **2011**, *2*, 1666–1676.

(8) Grayson, E. J.; Bernardes, G. J. L.; Chalker, J. M.; Boutureira, O.; Koeppe, J. R.; Davis, B. G. A coordinated synthesis and conjugation strategy for the preparation of homogeneous glycoconjugate vaccine candidates. *Angew. Chem. Int. Ed.* **2011**, *50*, 4127–4132.

(9) Boutureira, O.; Bernardes, G. J. L.; D'Hooge, F.; Davis, B. G. Direct radiolabelling of proteins at cysteine using [^{18}F]-fluorosugars. *Chem. Commun.* **2011**, *47*, 10010–10012.

(10) Fernández-González, M.; Boutureira, O.; Bernardes, G. J. L.; Chalker, J. M.; Young, M. A.; Errey, J. C.; Davis, B. G. Site-selective chemoenzymatic construction of synthetic glycoproteins using endoglycosidases. *Chem. Sci.* **2010**, *1*, 709–715.

(11) Chalker, J. M.; Lin, Y. A.; Boutureira, O.; Davis, B. G. Enabling olefin metathesis on proteins: chemical methods for installation of *S*-allyl cysteine. *Chem. Commun.* **2009**, 3714–3716.

(12) Bernardes, G. J. L.; Linderoth, L.; Doores, K. J.; Boutureira, O.; Davis, B. G. Site-selective traceless Staudinger ligation for glycoprotein synthesis reveals scope and limitations. *ChemBioChem* **2011**, *12*, 1383–1386.

(13) Boutureira, O.; D'Hooge, F.; Fernández-González, M.; Bernardes, G. J. L.; Sánchez-Navarro, M.; Koeppe, J. R.; Davis, B. G. Fluoroglycoproteins: ready chemical site-selective incorporation of fluorosugars into proteins. *Chem. Commun.* **2010**, *46*, 8142–8144.
